# DNA replication origins retain mobile licensing proteins

**DOI:** 10.1038/s41467-021-22216-x

**Published:** 2021-03-26

**Authors:** Humberto Sánchez, Kaley McCluskey, Theo van Laar, Edo van Veen, Filip M. Asscher, Belén Solano, John F. X. Diffley, Nynke H. Dekker

**Affiliations:** 1 Department of Bionanoscience, Kavli Institute of Nanoscience, Delft University of Technology, Delft, The Netherlands; 2 Chromosome Replication Laboratory, Francis Crick Institute, London, UK

**Keywords:** Single-molecule biophysics, Origin selection

## Abstract

DNA replication in eukaryotes initiates at many origins distributed across each chromosome. Origins are bound by the origin recognition complex (ORC), which, with Cdc6 and Cdt1, recruits and loads the Mcm2-7 (MCM) helicase as an inactive double hexamer during G1 phase. The replisome assembles at the activated helicase in S phase. Although the outline of replisome assembly is understood, little is known about the dynamics of individual proteins on DNA and how these contribute to proper complex formation. Here we show, using single-molecule optical trapping and confocal microscopy, that yeast ORC is a mobile protein that diffuses rapidly along DNA. Origin recognition halts this search process. Recruitment of MCM molecules in an ORC- and Cdc6-dependent fashion results in slow-moving ORC-MCM intermediates and MCMs that rapidly scan the DNA. Following ATP hydrolysis, salt-stable loading of MCM single and double hexamers was seen, both of which exhibit salt-dependent mobility. Our results demonstrate that effective helicase loading relies on an interplay between protein diffusion and origin recognition, and suggest that MCM is stably loaded onto DNA in multiple forms.

## Abstract (web summary)

Eukaryotic DNA replication is regulated to ensure copying of the genome (only) once per cell cycle. Here the authors, using optical trapping and confocal microscopy, demonstrate the dynamics of the origin recognition complex and subsequent intermediates that lead up to the loading of an MCM helicase onto DNA.

## Introduction

DNA replication in eukaryotes is a complex process whose control is critical for genome integrity and normal cell proliferation^1^. In the yeast *Saccharomyces cerevisiae*, specific DNA sequences are recognized by the origin recognition complex (ORC)^2^ and mark starting points for DNA replication before cells enter S-phase. These origins of replication were identified as autonomously replicating sequences (ARS) and consist of two elements: a strong binding site for ORC in the forward direction containing the AT-rich ACS (ARS consensus sequence) and B1 sequences, and a weak binding site in the reverse orientation (B2). More than 600 origins with different ORC affinities have been identified;^1,3,4^ only a subset of these origins are selected for active replication in any one S phase. Initiation requires the loading of two copies of an Mcm2-7 (MCM) hetero-hexameric helicase onto duplex DNA to form a double hexamer (DH)^5–8^; the recruitment of firing factors to assemble and activate the functional replicative helicase, CMG^9^; and the activity of replicative polymerases to perform bidirectional replication^10^. Furthermore, these origins are activated in a particular order in what has been referred to as the replication timing program^11^. In organisms from yeast to humans, many more MCM complexes are loaded onto chromatin than are used during S phase. Some of these excess MCMs likely act as “dormant replication origins”, which are important to rescue stalled replication forks and maintain genome integrity^12^. Recent evidence has suggested that there is a preference for MCM complexes inherited from the previous cell cycle to be used in replication^13^. Nonetheless, the “MCM paradox” is still unresolved.

The sequence of events that leads to the loading of the MCM DH onto DNA has been examined in biochemical^14,15^, single-molecule^16^, and cryo-electron microscopy (cryoEM) experiments^17–20^. First, ORC binds to origin DNA, encircling and bending it^21^. Cdc6 binds to ORC, creating a recruitment platform for Mcm2-7 and Cdt1. This recruitment reaction requires ATP binding by ORC, Cdc6, and MCM, and results in the formation of the Orc-Cdc6-Cdt1-Mcm2-7 (OCCM) complex^20^. After ATP hydrolysis, the Mcm2-7 ring closes^14,15,20^ and Cdt1 is released^16^. DH formation proceeds through sequential loading of each MCM^22^ by ORC molecules bound in opposing orientations^14,23^, in a process that may or may not involve the same ORC acting twice^24^.

What is less understood is how this sequence of events, including proper formation of the intermediates, is influenced by the motion of individual proteins on the DNA. A sliding helicase-loading intermediate has been suggested to explain the consequences of roadblock placement on the DNA^23,25^ and is required over short distances to permit the rebinding of ORC at the B2 site of the ARS1 origin following the establishment of an initial OCCM (ORC-Cdc6-Cdt1-MCM) intermediate^24^. It is known from bulk biochemical experiments that MCM DH are mobile, as they can diffuse off linear DNA^5^ and be displaced by RNA polymerase^26^ or CMG^9^. Such MCM DH dynamics could explain the observed uncoupling of replication initiation from the site of ORC binding^27^. However, whether the motion of proteins on the DNA has a role in origin recognition by ORC or in the formation of intermediates that precede the MCM DH has not been explored.

## Results

### ORC can bind DNA in a sequence-independent manner, but has a preference for the origin

**Fig. 1:**
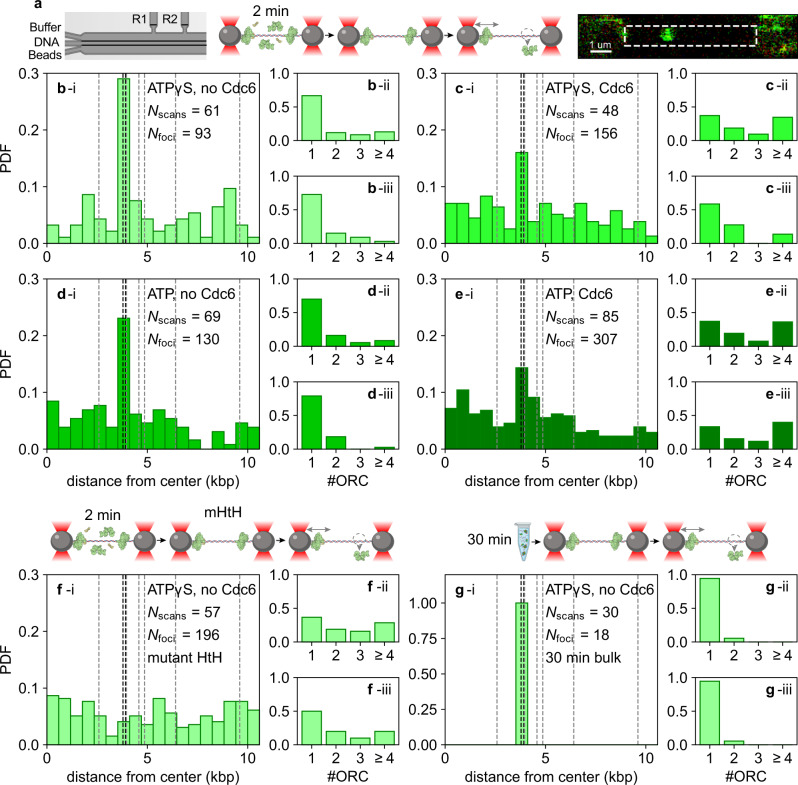
The spatial distribution of ORC is dominated by the origin. **a** From left to right: scheme of the flow cell, experimental workflow, and a representative image of labeled ORC bound to a single DNA molecule. The dashed square highlights the region used for data analysis. A DNA molecule is tethered to beads that are trapped in a dual-beam optical trap, allowing the DNA to be held under tension. When labeled ORC is introduced and binds to the DNA, it is excited by an integrated confocal laser scanning system (for further details, see Methods). **b**–**e** (i) Histograms of the spatial distribution of DNA-bound JF549-ORC following a 2 min-long incubation. (ii) Distribution of the stoichiometry of ORC foci everywhere on the DNA, and (iii) within 0.2 µm of the HtH origin (iii). Specific conditions are: **b** incubation of ORC in ATPγS; **c** ORC and Cdc6 in ATPγS; **d** ORC in ATP; **e** ORC and Cdc6 in ATP. **f** As in **b**, except that DNA contains the mHtH origin as described in Supplementary Fig. 7. **g** As in **b**, except that JF549-ORC is incubated with the 21.2 kbp DNA in bulk for 30 min before being introduced into the flow cell and imaged as in **b**–**e**. Bold dashed lines in the seventh bin from the left indicate the head-to-head (HtH) high-affinity ORC-binding sites. Faint dashed lines indicate near-cognate binding sites elsewhere on the DNA.

We took an in vitro single-molecule approach to examine the dynamics of the proteins involved in the ATP-dependent MCM-loading reaction (Supplementary Fig. 1). In these experiments, a 21.2 kbp biotinylated DNA molecule was tethered to strepatividin-coated beads in a dual-beam optical trap, allowing the DNA to be held under tension but without rotational constraint, as the optically trapped beads can freely reorient (Fig. 1a). To synthesize this DNA construct, we engineered a pSupercos1-lambda 1,2 plasmid with an artificial origin 6.7 kbp from one end. This “head-to-head” (HtH) origin consisted of two inverted high-affinity ORC binding sites spaced by 100 bp^23^. Sequence analysis showed that the plasmid also contained a number of endogenous potential binding sites for ORC^3^ (Supplementary Fig. 2a, b).

To visualize ORC, we labeled the N-terminus of the Orc3 subunit with a JF549 fluorophore via a HaloTag (Methods) and confirmed that it could load MCM in bulk assays (Supplementary Fig. 2c, d). We then incubated optically trapped DNA under near-zero force for 2 min in a reservoir containing 5 nM JF549-ORC before moving to a separate, protein-free channel of the microfluidic chip for imaging under a force of 2 pN. DNA-bound JF549-ORC was detected as a bright fluorescent spot (focus) (Fig. 1a, right panel). Higher forces during incubation led to a decreased number of such foci, including at the origin, and thus were not considered further (Supplementary Fig. 3a). DNA-bound ORC molecules could be removed via a high-salt wash (HSW), as expected from previous biochemical analysis (Supplementary Fig. 3b, c)^28,29^.

By probing the overall fluorescence intensity of DNA-bound ORC after defined waiting times in the dark, we noted an initial phase of rapid unbinding with a mean lifetime of 8.6 s, followed by a slower phase of unbinding with a mean lifetime of 1278 s (Supplementary Fig. 3d). Short-lived ORC–DNA interactions lasting <10 s have been previously attributed to those occurring at non-origin DNA^22^, whereas the longer lifetime is consistent with the slow turnover of ORC bound to the origin^30^.

Next, we examined the spatial distribution of ORC foci on the DNA. As the DNA has two possible orientations in the optical trap, the position of the origin is not known a priori. Therefore, we represent the ORC spatial distribution, and all other spatial distributions in this paper, in terms of radial distance from the midpoint of the DNA. Each histogram bin of 0.59 kbp contains the average of the occupancies of two symmetrically located stretches of DNA on each side of the midpoint. Accordingly, the “HtH bin” contains the average of the occupancy of the origin-containing bin and the region of DNA 6.7 kbp from the other end of the substrate (see Methods for further details). Following a 2 min incubation with 5 nM ORC, fluorescence foci (filtered to remove foci containing >10 ORC, which could represent aggregates; see Methods) were present throughout the DNA molecule, but were clearly overrepresented in the bin containing the HtH (Fig. 1), consistent with preferred binding of ORC at the origin. This origin preference was observed in all biochemical conditions tested (ORC alone in the presence of ATP or the slowly hydrolyzable ATP analog ATPγS, and ORC and Cdc6 jointly incubated in a buffer containing ATPγS or ATP). We observed a more pronounced origin preference in the presence of ATPγS (compare Fig. 1b, c to Fig. 1d, e) and the absence of Cdc6 (compare Fig. 1b, c to Fig. 1c, e). Of note, the preferred binding of ORC at the origin was strongly dependent on the sequence characteristics of the HtH origin: mutation of the origin (mHtH) eliminated all signatures of preferential binding, irrespective of the biochemical condition (Fig. 1f and Supplementary Fig. 4a–d).

Next, we examined the ORC stoichiometry in these experiments by counting the number of bleaching steps, assessed and validated using dCas9 tagged and labeled in an identical fashion to ORC (Supplementary Fig. 5). We found that in the absence of Cdc6, the population was dominated by individual ORC molecules (panels ii and iii in Fig. 1b–e), with no increase in the vicinity of HtH (compare panels iii to ii in Fig. 1b–e). The presence of Cdc6 resulted in higher stoichiometries (compare Fig. 1c, e to Fig. 1b, d), suggesting enhanced binding, but again not in a manner specific to the origin (compare panels ii and iii in Fig. 1c, e).

We also examined the lifetimes of individual ORC molecules on the DNA. These lifetimes were measured by tracking the spatial coordinates of foci containing a single ORC molecule at a frame rate of 0.6 s/scan until the signal from the fluorophore disappeared. The lifetimes tended to be short, between 5 and 20 s (Supplementary Fig. 3e). As the lifetime of the JF549 dye under these imaging conditions is 60.6 s (assessed with dCas9-JF549 bound to identical DNA molecules; Supplementary Fig. 5b, panel iv), one would expect ~72% of the JF549-ORC fluorophores to bleach at times longer than 20 s. Thus, we deduced that most of the individual ORC molecules dissociated from the DNA, which is consistent with the initial phase of rapid unbinding of ORC from the DNA deduced from the quantification of the total ORC intensity after selected time delays in the dark, as described above (Supplementary Fig. 3d). Despite this, greater stability of ORC at the origin could be detected, as the lifetime of ORC at the origin was nearly twofold longer than elsewhere in the presence of ATP (Supplementary Fig. 3f).

To probe origin recognition by ORC in an alternative manner, we incubated the same DNA with 5 nM ORC in bulk for 30 min. We then trapped these pre-incubated DNA molecules and imaged ORC as described above. Contrary to our observations following 2-min in-flow-cell incubations (Fig. 1b–e), we observed either DNA molecules devoid of ORC, or DNA molecules in which ORC was exclusively localized to the HtH bin (Fig. 1g and Supplementary Fig. 4e–h), emphasizing the stability of ORC binding to the origin compared to all other DNA sequences in our 21.2 kbp DNA. However, the longer incubation did not increase the stoichiometry at the HtH (Supplementary Fig. 4e-h), despite its two ORC binding sites. Indeed, subsequent experimentation indicated that an incubation time in bulk of 5 min yielded similar results. Consistently, when experiments with a 30-min incubation time were repeated on DNA molecules containing the mutated origin (mHtH), only DNA molecules devoid of ORC were observed (Supplementary Fig. 4i). We also repeated a subset of these experiments that probe the spatial distribution of ORC with ORC labeled with the dye JF646, but found minimal difference in either spatial distribution or stoichiometry relative to JF549-ORC, indicating that the results are independent of the fluorophore employed (Supplementary Fig. 6). Overall, we conclude that the behavior of ORC in this single-molecule context is broadly consistent with earlier biochemical and biophysical findings.

### ORC performs a linear target search on DNA to find the origin

**Fig. 2:**
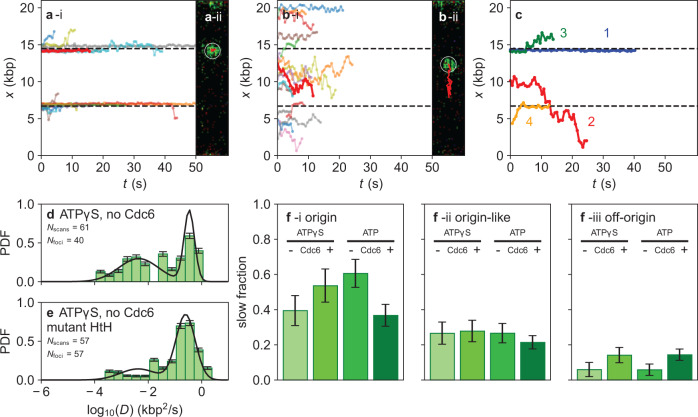
ORC molecules exhibit diffusive motion that is halted at origins. **a**, **b** (i) Sample time traces and (ii) scan images that illustrate the observed motion of JF549-ORC initially localized **a** within 0.2 µm of the HtH origin or **b** in any other location. Traces shown represent 3% (randomly selected) of traces from all four biochemical conditions described in Fig. 1. **c** (i) Sample time traces and (ii) scan images that illustrate the four main types of motion observed for JF549-ORC: (1) static; (2) diffusive; (3) static and then diffusive; (4) diffusive and then static. **d** Histogram of the diffusion constants of ORC incubated in the presence of ATPγS. Only foci containing 1 or 2 ORC were considered. The two populations of diffusion constants fit to log-normal distributions (solid black lines), taking into account the error bars derived from bootstrapping the data set 100 times. This yields populations with mean ± SEM of 0.06 ± 0.04 kbp^2^ s^−1^ (59% of the distribution) and 0.97 ± 0.09 kbp^2^ s^−1^ (*p* < 0.0001 by one-way ANOVA). **e** As in **d**, except that the DNA contains the mHtH origin described in Supplementary Fig. 7. Here, fitting yields populations with mean ± SEM 0.05 ± 0.03 kbp^2^ s^−1^ (26%) and 0.88 ± 0.09 kbp^2^ s^−1^ (*p* < 0.0001 by one-way ANOVA). **f** Quantification of the percent of ORC molecules initially bound in a given location, which go on to display slow diffusion: (i) ORC molecules initially bound within 0.2 µm of the HtH origin; (ii) ORC molecules initially bound within 0.2 µm of origin-like sequences; (iii) ORC molecules initially bound elsewhere. Error bars represent the error of sample proportion, sqrt(*p*(1−p)/*n*), where *p* is the proportion of a sample in a given population, and *n* is the sample size.

When we tracked the position over time of ORC molecules initially located within 0.2 µm of the HtH bin (Fig. 2a–i, showing 3% of all traces randomly selected), we observed that the position of many of these ORC molecules hardly changes over time, within experimental error. The microscope image of one such ORC molecule, with its tracked spatial evolution indicated in red, is shown in panel ii. Conversely, many ORC molecules that were initially located outside the HtH (Fig. 2b–i, showing 3% of all traces randomly selected, and panel ii, showing one example image), explored the local DNA environment in an apparently random manner. These primary categories of ORC dynamics are highlighted as examples 1, 2 in Fig. 2c. However, we also observed ORC molecules that were initially static in the HtH bin but then started to explore nearby regions of the DNA (example 3 in Fig. 2c; also apparent in several traces in Fig. 2a–i), and molecules that were dynamic but became static when they entered the HtH bin (example 4 in Fig. 2c). Movies illustrating these types of behavior are shown in Supplementary Movies 1, 2.

To quantify this behavior, we computed the mean-square displacement for each ORC molecule as a function of time interval and extracted a diffusion constant from a linear fit. A histogram showing all the fitted diffusion constants is shown in Fig. 2d (note the log scale) for ORC incubated in buffer containing ATPγS. We observed a wide spread of diffusion constants, ranging from 10^−5^ to 10^0^ kbp^2^ s^−1^. Nevertheless, we could clearly fit one part of the distribution to a lower diffusion constant of 0.06 ± 0.04 kbp^2^ s^−1^ (mean ± SEM), presumably reflecting slowly moving or static ORC molecules, and another part of the distribution to a higher diffusion constant of 0.97 ± 0.09 kbp^2^ s^−1^, reflecting more rapidly moving ORC molecules (*p* < 0.0001 by one-way ANOVA). ORC molecules that displayed both static and diffusive behavior correspond to intermediate values of the diffusion constant.

A similar bimodal distribution of diffusion constants for ORC was observed for all biochemical conditions (Supplementary Fig. 7a–d), although the presence of Cdc6 led to an increase in the population of ORC molecules with fast or intermediate diffusive behavior (Supplementary Fig. 7b, d). This was also the case for ORC labeled with JF646, indicating that the mobility was unaffected by the particular fluorophore employed (Supplementary Fig. 8a, b). On DNA molecules with the mHtH sequence, the subpopulation fitting to lower values of the diffusion constant was reduced in abundance and no longer spatially correlated with the origin site, as shown in Fig. 2e for ORC incubated in buffer containing ATPγS (and in Supplementary Fig. 7g–m for all other biochemical conditions).

dCas9-JF549 statically bound to identical DNA molecules yielded a singly peaked distribution of diffusion constants with a mean of 0.01 ± 0.01 kbp^2^ s^−1^ (Supplementary Fig. 5d–ii), which provides a lower limit on the measurable diffusion constant under these imaging conditions. We confirmed that ORC molecules bound to the origin following a 30-min incubation in bulk (Fig. 1 g) were also mainly slow or static, with an average diffusion constant of 0.01 ± 0.01 kbp^2^ s^−1^ (Supplementary Fig. 7n and 8c). Based on these findings, we conclude that the slower subpopulations associated with mean diffusion constants ~10^−2^ kbp^2^ s^−1^ reflect ORC molecules that are largely static on the DNA.

We next asked whether there was a correlation between ORC mobility and DNA sequence. We determined what fraction of ORC molecules initially localized in the HtH bin (see Fig. 1) were classified as part of the static subpopulation. This fraction was relatively independent of the biochemical conditions tested, ranging between 40 and 60% (Fig. 2f–i). For ORC molecules initially localized in bins containing potential binding sites for ORC, this fraction was reduced to 20–30% (Fig. 2f–ii), whereas in bins containing no such sequences, the fraction was <20% (Fig. 2f–iii). This establishes a correlation between localization of ORC molecules at the origin and reduced mobility. Jointly, these results suggest a scenario in which the ORC binds aspecifically to much of the DNA, yet can locate its preferred binding site at the origin by linearly scanning the DNA. Once at the origin, ORC mobility is reduced, presumably allowing MCM recruitment to preferentially proceed from there.

### Accumulation of loading intermediates at the origin occurs in the presence of fast-moving MCMs

**Fig. 3:**
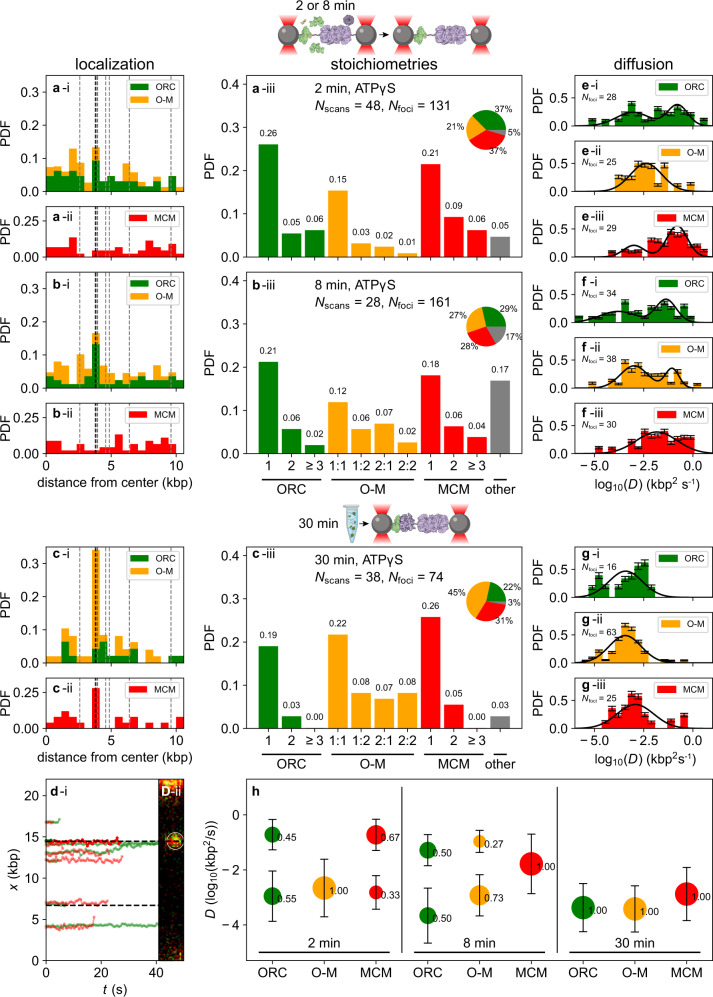
Spatial distribution, stoichiometry, and diffusive behavior of loading intermediates observed in ATPγS. **a** Histograms of the spatial distribution of fluorescent foci after a 2-min incubation in the flow cell. (i) Stacked bins representing foci containing 1 or 2 ORC (green) or colocalized ORC and MCM (orange), and (ii) foci containing 1 or 2 MCM (red) molecules. The overall stoichiometry distributions of these foci are shown in panel (iii). The gray bin labeled “other” accounts for all colocalized foci where either the ORC or MCM stoichiometry is 3 or higher. **b** As in **a**, but following an incubation time of 8 min. **c** As in** a** and **b**, but following a 30-min incubation in bulk, ×1000 dilution, and introduction into the flow cell. **d** Sample time traces and corresponding image to illustrate the motion of colocalized JF549-ORC (green) and JF646-MCM (red) acquired at a frame rate of 0.6 s. **e**–**g** Histograms of the diffusion constants of the loading intermediates shown in **a**–**c**. The populations of diffusion constants fit log-normal distributions (solid black lines), taking into account the error bars derived from bootstrapping the data set 100 times. **e** After a 2-min incubation in the flow cell, the fitted diffusion coefficients are (mean ± SEM, in units of kbp^2^ s^−1^): ORC: 0.02 ± 0.04 and 1.0 ± 0.4 (*p* = 0.008 by one-way ANOVA); O-M: 0.07 ± 0.06; MCM: 0.009 ± 0.005 and 1.0 ± 0.3 (*p* = 0.04 by one-way ANOVA). **f** After an 8 min incubation, the diffusion coefficients are (mean ± SEM, in kbp^2^ s^−1^): ORC: 0.01 ± 0.02 and 0.28 ± 0.09 (*p* = 0.007 by one-way ANOVA); O-M: 0.012 ± 0.007 and 0.39 ± 0.09 (*p* < 0.0001 by one-way ANOVA); MCM: 0.9 ± 2.3. **g** After the bulk 30-min incubation and introduction into the flow cell, the diffusion coefficients are (mean ± SEM, in kbp^2^ s^−1^): ORC: 0.008 ± 0.009; O-M: 0.006 ± 0.003; MCM: 0.04 ± 0.06. **h** Summary of the fitted diffusion constants in **e**–**g** for different incubation times and conditions (e.g., in-flow-cell or bulk).

To test this hypothesis, we added JF646-labeled MCM to the reactions. MCM was labeled through the introduction of a HaloTag on the N-terminus of its Mcm3 subunit, and JF646-MCM performed normally in a bulk loading assay in conjunction with JF549-ORC (Supplementary Fig. 9). We first performed single-molecule experiments in which JF549-ORC, Cdc6, and JF646-Mcm2-7/Cdt1 were loaded into the flow cell in a buffer containing ATPγS and visualized there after a 2-min or 8-min incubation (Fig. 3a, b). In these experiments, recruitment of MCM to the DNA depended on the joint presence of ORC and Cdc6 in the flow cell (Supplementary Fig. 10a). As in the case of ORC alone, most of the DNA-bound proteins could be removed via an HSW in the flow cell (compare panels i and ii in Supplementary Fig. 10b; quantification in Supplementary Fig. 10c).

The spatial distributions and relative stoichiometries of ORC and MCM following a 2-min incubation are shown in Fig. 3a (the stoichiometry of MCM is also assessed by counting the number of bleaching steps; Supplementary Fig. 10d). In these spatial distributions, we included ORC foci with stoichiometries of 1 or 2 (green; 37% of the population), ORC:MCM foci with stoichiometries 1:1, 1:2, 2:1, or 2:2 (orange; 21% of the population), and MCM foci with stoichiometries of 1 or 2 (red; 37% of the population) (Fig. 3a, panel iii). Higher-order stoichiometries, collected in the bin labeled “other”, were rare, comprising only 5% of the population. As in the experiments performed with ORC alone (Fig. 1), the spatial distribution of ORC was broad (green bins in Fig. 3a–i). ORC continued to bind preferentially in the vicinity of the HtH bin, though the peak was less pronounced by a factor of two than in directly comparable experiments lacking MCM (Fig. 1b–i). The spatial distribution of colocalized ORC-MCM (orange bins in Fig. 3a–i) was fairly uniform across the DNA and did not display noticeable features at the origin. Summing these two populations resulted in a spatial profile that was broadly peaked in the vicinity of the origin (Fig. 3a–i). Interestingly, we observed a substantial population of MCM foci that were not colocalized with ORC, and that were found broadly distributed over the DNA (Fig. 3a–ii). As these MCMs were recruited to the DNA in an ORC- and Cdc6-dependent manner, we speculate that MCM can decouple from ORC while remaining bound to the DNA. It is also interesting to speculate that their presence on the DNA inhibits diffusion by ORC, and hence reduces its presence at the origin (Fig. 3a–i).

After an 8-min incubation time in the flow cell, the ORC-MCM population (27%) increased slightly at the expense of the ORC-only (29%) and MCM-only (28%) populations (Fig. 3b–iii). We observed clearer features of an overall ORC preference for the origin (green bins in Fig. 3b–i), whereas the spatial distribution of colocalized ORC-MCM remained relatively uniform (stacked orange bins in Fig. 3b–i). Compared with the experiments performed with a 2-min incubation time, the sum of these populations more clearly showed an overall preference of ORC for the origin. The population of MCM foci that were not colocalized with ORC continued to display a uniform distribution across the DNA (Fig. 3b–ii).

To further enrich the population of potential loading intermediates (ORC-MCM foci), we performed a bulk incubation of the loading proteins in ATPγS for 30 min and examined the result in the flow cell (Fig. 3c). The ORC-MCM population (45%) again increased at the expense of the ORC-only (22%) and MCM-only (31%) populations (Fig. 3c–iii). Notably, this increase in the colocalized population did not simply result from greater crowding of proteins on the DNA (the population of foci with higher-order stoichiometry accounted for only 3% of foci detected). Compared with the shorter in-flow-cell incubations, a distinct ORC population near the origin was no longer seen (green bins in Fig. 3c–i). However, as the ORC-only population was very sparse and the population of colocalized ORC-MCM displayed a pronounced peak in the HtH bin (stacked orange bins in Fig. 3c–i), ORC as a whole retained an overall preference for the origin. Unexpectedly, the spatial distribution of MCM foci not colocalized with ORC also showed a pronounced peak in the HtH bin (Fig. 3c–ii), even in the absence of ATP hydrolysis. As suggested above, this may result from decoupling of ORC from some MCMs, which remain bound to the DNA.

We next examined the mobility of colocalized ORC-MCM, potential intermediates in MCM recruitment. Following 2-min or 8-min incubation in the flow cell, ORC molecules that were spatially separated from MCM continued to show both fast and slowly diffusive populations (panels i in Fig. 3e, f). A similar range of diffusion coefficients was observed for colocalized ORC-MCM (Fig. 3e–ii, f–ii), although the distribution in Fig. 3e–ii could only be fit to a single, slowly diffusing population (Fig. 3h). Traces showing the colocalization (and movement) of ORC and MCM over extended periods of time are depicted in Fig. 3d (see also Supplementary Movie 3). Interestingly, following short incubation times, nearly all recruited MCM molecules that were not colocalized with ORC were highly mobile, with a mean diffusion constant of 1.0 ± 0.3 kbp^2^ s^−1^ (mean ± SEM) (Fig. 3e–iii) or 0.9 ± 2.3 kbp^2^ s^−1^ (Fig. 3f–iii; quantified in Fig. 3h). This high mobility may account for its relatively uniform spatial distribution following short incubations in the flow cell (Fig. 3a–ii, b–ii). Following bulk incubation for 30 min and introduction into the flow cell, we observed only slowly diffusing populations for all three species (panels i–iii in Fig. 3g; quantified in Fig. 3h; reference experiments with dCas9-JF646 and dCas9-JF549 in Supplementary Fig. 5d). For the few ORC molecules that were not colocalized with MCM, this was expected based on our experiments with ORC alone (Supplementary Fig. 7n); for colocalized ORC-MCM and MCM alone, this observation reinforced the notion that the origin retains mostly slowly diffusing intermediates, whereas more rapidly diffusing intermediates can be lost through diffusion off the linear DNA ends during bulk incubation.

### Salt-stable loaded MCM single and DHs are slowly diffusive

We next assessed how these dynamics impacted the evolution of intermediates toward MCM DH formation in the presence of ATP. JF549-ORC, Cdc6, and JF646-Mcm2-7/Cdt1 were loaded into the flow cell in a buffer containing ATP and visualized there. As in the experiments in ATPγS, MCM recruitment and/or loading was absolutely dependent on the presence of ORC (Supplementary Fig. 11). Quantification of the relative stoichiometries of ORC, colocalized ORC-MCM, and MCM is shown in Supplementary Fig. 12a–c. We observed that following a 2-min incubation, the fraction of the population represented by colocalized ORC-MCM was similar in ATP (21%, orange data in Supplementary Fig. 12a) and ATPγS (21%), but it decreased to 15% after 16 min in ATP (orange data in Supplementary Fig. 12b). This was in contrast with the experiments performed in ATPγS, where the fractional population of colocalized ORC-MCM increased to 27% after only 8 min. Foci containing only MCM comprised 53% of the population following a 2-min incubation, and 45% of the population following a 16-min incubation (red data in Supplementary Fig. 12a, b, respectively). As the incubation time increased, more of the MCM-only foci contained two or more MCMs rather than just a single MCM (Supplementary Fig. 12a, b), a trend that was not observed in ATPγS (panels iii in Fig. 3a, b). Similar analysis following a 30-min incubation in bulk in the presence of ATP showed that these trends were accentuated: the colocalized ORC-MCM population was nearly absent at 5% (orange data in Supplementary Fig. 12c), and the MCM-only population was highly dominant at 86% (red data in Supplementary Fig. 12c). Within the MCM population, 52% of foci contained two or more MCMs (Supplementary Fig. 12c), as compared with 5% in ATPγS (panel iii in Fig. 3c). The evolution of the MCM-containing species towards an increasing fraction of MCM DH as a function of incubation time is plotted in Supplementary Fig. 12d, confirming the trends found in electron microscopy (EM) experiments^24^.

**Fig. 4:**
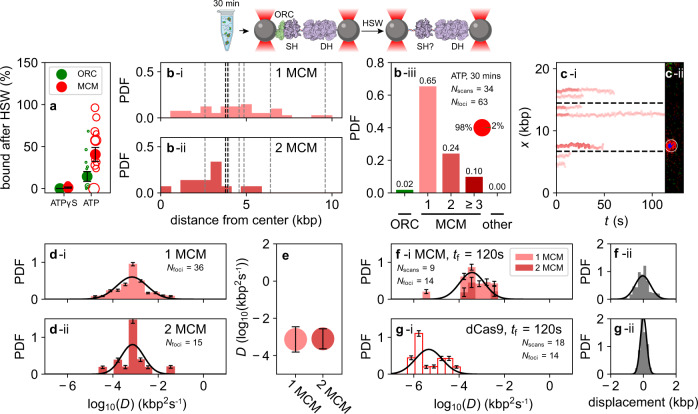
Spatial distribution, stoichiometry, and diffusive behavior of MCM in ATP after HSW. **a** The fraction of ORC and MCM complexes that survive an in-situ HSW following incubation in ATPγS (left) or ATP (right). Percentages are the ratio of the total fluorescence of ORC (green) or MCM (red) before and after the HSW. Open circles are individual measurements, while the filled dots and error bars are the sample mean and S.D., respectively. In ATPγS (green), *N* = 6, whereas in ATP, *N* = 11. **b** (i–ii) The spatial distributions of the fluorescent foci, and (iii) the overall stoichiometry distribution. The gray bin labeled “other” accounts for all foci with ORC or MCM stoichiometries higher than 3. **c** Images and sample time traces that illustrate the motion of foci initially containing one (light red) or two (red) MCM. **d** Histograms of the diffusion constants of foci containing a single MCM (i) or two MCM (ii). Log-normal fits to the distributions of single (double) MCMs yield mean ± SEM of 0.006 ± 0.002 kbp^2^ s^−1^ (0.004 ± 0.001 kbp^2^ s^−1^), taking into account the error bars derived from bootstrapping the data set 100 times. **e** Summary plot of the diffusion constants derived from the data in **d**. **f** (i) Histogram of the diffusion constants of foci containing a single MCM (light red) or two MCM (red) imaged in high-salt buffer at an acquisition frequency of one frame every 120 s. The fitted diffusion coefficient was 0.0023 ± 0.0009 kbp^2^ s^−1^ (mean ± SEM). (ii) Histogram of the net displacements observed for the same MCM molecules as in (i). **g** (i) Histogram of the diffusion constants for foci containing dCas9-JF646 imaged in high-salt buffer at the same reduced acquisition frequency, with diffusion coefficient (3 ± 1) × 10^−5^ kbp^2^ s^−1^ (mean ± SEM). By one-way ANOVA, the distributions in **f** (i) and **g** (i) are statistically distinct (*p* < 0.0001). (ii) Histogram of the net displacements observed for the same dCas9 molecules as in (i).

Ultimately, the MCMs that are stably loaded onto DNA are those that resist a HSW following ATP hydrolysis. To probe their spatial positioning, stoichiometry, and dynamics, we again incubated the loading proteins with DNA for 30 min in bulk in the presence of ATP and performed a HSW in the flow cell before imaging (Fig. 4). We did not perform experiments with both incubation and HSW within the flow cell, because under these conditions the HSW sometimes increased the number of proteins bound to the DNA, possibly a result of bead-adhered proteins being released onto the tethered DNA. Under these conditions, most ORC proteins dissociated from the DNA following HSW, but many of the MCM molecules remained DNA-bound (Fig. 4a). This contrasted with the result of experiments performed in ATPγS, where both ORC and MCM molecules dissociated following HSW (Supplementary Fig. 10c), and was fully consistent with bulk biochemical loading assays (Supplementary Fig. 2d; Supplementary Fig. 9b). Indeed, following ATP hydrolysis and HSW, MCM molecules represented 98% of the molecules remaining on the DNA (Fig. 4b, panel iii).

We plotted the spatial distributions of the MCM molecules that survived a HSW and observed them to be symmetrically distributed near the origin (Fig. 4b–i, ii). The spatial distribution of foci containing two MCM (panel ii) was more sharply peaked than the distribution of foci containing one MCM (panel i). Overall, the foci showed a stoichiometry distribution in which 65% of the foci contained a single MCM, and 24% of the foci contained 2 MCM (Fig. 4b–iii). The ratio of single to double MCM following ATP hydrolysis and HSW was similar to previous single-molecule observations^22^. It is very likely that foci containing two MCM indeed reflect the presence of bona fide DHs (as opposed to two individual Mcm2-7 complexes present within the same diffraction-limited focus) because a focus containing two MCM is maintained as a single unit over an extended period of time (Fig. 4c), a conclusion reinforced by experiments on longer timescales described below.

We next investigated the mobility of MCMs that survive the HSW following ATP hydrolysis. Diffusion analysis of foci containing either 1 or 2 MCM revealed a singly peaked, slowly diffusive distribution in both cases, with no discernible quantitative difference between them (Fig. 4d–i, ii; quantification in Fig. 4e; reference experiment with dCas9-JF646 in Supplementary Fig. 5d–i). Under these conditions, MCM movement was indistinguishable from the lower limit measured using dCas9-JF646. However, published bulk biochemical experiments find a half-life of 10 min for loaded MCM on linear 1 kbp DNA fragments in high-salt buffer^5^, so loaded MCM should be diffusive on longer timescales. To specifically address this, we imaged JF646-MCM at a rate of one frame every 120 s rather than every 0.6 s in high-salt buffer (Fig. 4f) and compared it to dCas9-JF646 (Fig. 4g). Under these conditions, the motion of molecules could be followed for thousands of seconds. The diffusion coefficient of dCas9 was (3 ± 1) × 10^−5^ kbp^2^ s^−1^ (Fig. 4g–i), whereas MCM diffused two orders of magnitude faster, at 0.0023 ± 0.0009 kbp^2^ s^−1^ (Fig. 4f–i). The distribution of net displacements of MCM (Fig. 4f−ii; *σ* = 0.47 kbp) was also much wider than the (presumably experimental noise-dominated) displacement distribution of dCas9 (Fig. 4g–ii; *σ* = 0.2 kbp). These results indicate that stably loaded MCMs, whether single or DH in form, are slowly but distinctly mobile (Supplementary Movies 4,5).

## Discussion

### DNA-bound ORC performs a linear target search for the origin

In yeast, origins of replication are known to be sequence-specific in a manner that is dictated by the sequence preference of ORC^2^. Our results indicate that yeast ORC can locate these sequences through a process of linear diffusion on the DNA (Fig. 2) that is slightly enhanced by association with Cdc6 in the presence of ATP (Supplementary Fig. 7j), following sequence-independent initial binding (Fig. 1). Once at the origin, the mobility of ORC is substantially reduced. For comparison, another ring-shaped protein involved in DNA replication, human PCNA, diffuses at 2.24 kbp^2^ s^−1^ (1.16 µm^2^ s^−1^)^31^ under similar buffer conditions to ours (150 mM potassium glutamate), approximately two orders of magnitude higher than what we measure for yeast ORC in its fast-diffusing mode. That yeast ORC would have a lower diffusion constant would make sense in the context of its need to probe the DNA sequence.

The diffusive motion of ORC has observable consequences in our experiments. For example, the nearly absolute sequence specificity observed following bulk incubation of ORC with linear DNA and subsequent dilution for introduction into the flow cell (Fig. 1g) likely results not only from more rapid dissociation of ORC from non-origin DNA, including during sample handling (Supplementary Fig 3e and ref. ^30^), but also from sliding off the exposed ends of the DNA. Similar dilution of ORC-bound tethered DNA in the flow cell did not remove ORC molecules bound outside the origin as completely (Supplementary Figure 4j), suggesting that ORC’s encirclement of DNA reduced the total rate of dissociation from DNA.

In higher eukaryotes, ORC does not exhibit sequence specificity in its binding, and would apparently have no need of target search via diffusion. Nevertheless, given the importance of DNA bending by ORC for subsequent recruitment of MCM, it has been suggested that diffusion of ORC may be required to find sufficiently bendable DNA^32^. Diffusion could particularly facilitate the search for binding sites in regions of actively transcribed DNA where nucleosome occupancy is reduced^33^. Nucleosomes themselves have been found to act as potential roadblocks for MCM diffusion^24^, and it will be interesting to examine their roles in either directly limiting or locally targeting the diffusion of ORC on DNA.

### Loading intermediates

Our experiments in ATPγS indicate that ORC- and Cdc6-dependent MCM recruitment to DNA occurs readily. At early time points, colocalized ORC-MCM intermediates are found throughout much of the DNA (Fig. 3a, b). Although it does not appear that colocalized ORC-MCM intermediates are exclusively formed at slowly diffusive ORC molecules, the slowly diffusive colocalized ORC-MCM population nonetheless exceeds the rapidly diffusive population (Fig. 3e–g). At these early time points, we also find isolated MCM on much of the DNA (Fig. 3a, b), which are recruited in an ORC- and Cdc6-dependent manner but can apparently dissociate from their loaders while remaining on the DNA. These MCM molecules may impact the ability of ORC to locate the origin via diffusion by acting as intervening obstacles on the DNA, as we observe that the preference of ORC for the origin takes longer to establish in the presence of MCM (Fig. 3a, b). With increased incubation time, we do observe that first ORC, then colocalized ORC-MCM intermediates (and even isolated MCM), establish a preference for the origin (Fig. 3a–c). We have considered whether the preference for the origin displayed by colocalized ORC-MCM and MCM following 30-min incubation in bulk (Fig. 3c) simply resulted from diffusion of mobile ORC-MCM and MCM off the ends of the linear DNA employed in these experiments^5^. Although such diffusion does likely occur, (Fig. 3e–g) and as such will contribute to the apparent specificity at the origin, we additionally note that few colocalized ORC-MCM and MCM are found elsewhere, e.g., towards the center of the DNA (Fig. 3c). Thus, the presence of colocalized ORC-MCM and MCM at the origin likely reflects preferred recruitment there, mirroring the pronounced preference of ORC for the HtH bin under similar conditions (Fig. 1g; Supplementary Fig. 4e–h).

### Implications of the mobility of loaded MCM

In the presence of ATP, we find a gradual reduction over time of ORC-MCM intermediates on the DNA, and a corresponding increase in loaded MCM (Supplementary Fig. 12), as expected from cryoEM experiments^24^. Many of these MCM are salt-stable (Fig. 4a), contrasting with the case in ATPγS, and their spatial distribution is peaked near the origin (Fig. 4b), suggesting preferential loading there. Notably, we find a substantial presence of MCM SH in addition to the expected population of MCM DH (Fig. 4b–i, iii).

At first sight, both of these populations appear similarly static on the DNA (Fig. 4d, e), but closer investigation reveals clear mobility relative to an immobile standard such as dCas9 (Fig. 4f, g). The large abundance of the MCM SH species suggests a lack of coordination in the loading of MCM hexamers and may indicate, given their long lifetimes on the DNA, a requirement for mechanisms that remove unproductive MCM SH. It also suggests that at least some of the “MCM paradox”—that many more MCM is loaded than are actually used—may be owing to loaded SH species. Whether these SH species play any role in replication or other nuclear functions is an interesting question for further investigation.

The activation of CMG helicase relies on the formation of an MCM DH^9^, and how such MCM DH are formed has been a matter of debate. It seems likely that the loading pathway recently suggested by EM experiments, in which ORC molecules bound to opposite termini of a single MCM hexamer cooperate to load the second hexamer in the proper orientation for DH formation^24^, is the predominant productive pathway. Yet given the large proportion of single MCMs that resist HSW (Fig. 4 and ref. ^22^), a pathway in which single MCMs loaded by distinct ORC molecules could encounter one another via diffusion and lead to DH formation remains a possibility. This could account for the functionality in vivo of artificial origins with inverted ORC-binding sites spaced up to 400 bp^23^. It has also long been known that an excess of MCMs relative to those that are necessary to carry out replication during S-phase are loaded onto DNA^34^. These MCMs have been associated with the ability of cells to restart replication during replicative stress^35^, and more recently, linked to the formation of topologically associated domains^36^. Our experiments indicate that both SH and DH forms of MCM can be stably maintained on the DNA and could contribute to such diverse roles.

## Methods

### Biological materials

#### Protein purification

*Cdc6*. *Saccharomyces cerevisiae* Cdc6 protein expression was induced in BL21-CodonPlus(DE3)-RIL cells (Agilent #230245) transformed with pGEX-6P-1 wt GST-cdc6 with 400 µM IPTG for 16 h at 16°C. Cells were harvested in Cdc6 lysis buffer (50 mM K_X_PO_4_ pH 7.6, 150 mM KOAc, 5 mM MgCl_2_, 1% Triton X-100, 2 mM ATP, cOmplete^TM^ EDTA-free Protease Inhibitors (Sigma-Aldrich #5056489001), and 1 mM DTT) and sonicated in a Qsonica Q500 sonicator for 2 min with cycles of 5 s and 5 s off and an amplitude of 40%. After centrifugation, Cdc6 protein was purified from the supernatant by incubating for 1 h at 4°C with glutathione beads Fastflow (GE Healthcare #17-5132-02). The beads were washed 20 times with 5 ml Cdc6 lysis buffer, and Cdc6 was released from the beads by digestion with Precision protease (GE Healthcare #27-0843-01) at 4°C for 16 h. Subsequently, the Cdc6 eluate was diluted with Cdc6 dilution buffer (50 mM K_X_PO_4_ pH 7.6, 5 mM MgCl_2_, 0.1% Triton X-100, 2 mM ATP, and 1 mM DTT) to a final KOAc concentration of 75 mM and incubated with hydroxyapatite Bio gel HTP (Bio-rad #130-0402) for 45 min at 4°C. The beads were washed five times with Cdc6 wash buffer (50 mM K_X_PO_4_ pH 7.6, 75 mM KOAc, 5 mM MgCl_2_, 0.1% Triton X-100, 2 mM ATP, and 1 mM DTT), then washed five times with Cdc6 rinse buffer (50 mM K_X_PO_4_ pH 7.6, 150 mM KOAc, 5 mM MgCl_2_, 15% glycerol, 0.1% Triton X-100, and 1 mM DTT). Then Cdc6 was eluted from the column in 1-ml fractions with Cdc6 elution buffer (50 mM KXPO4 pH 7.6, 400 mM KOAc, 5 mM MgCl_2_, 15% glycerol, 0.1% Triton X-100, and 1 mM DTT). Finally, fractions containing Cdc6 were pooled, dialyzed twice for 1 h against Cdc6 dialysis buffer (25 mM HEPES-KOH pH 7.6, 100 mM KOAc, 10 mM MgOAc, 10% glycerol, and 0.02% NP-40 substitute) in a 10 kDa cutoff Slide-A-Lyzer Cassette (Thermo Scientific #66380), and concentrated in an Amicon Ultra-4 Ultracell 30 kDa centrifugal filter (Merck-Millipore #UFC803024). Aliquots were snapfrozen and stored at −80°C. The protein concentration was determined with Bio-Rad Protein Assay Dye Reagent (Bio-rad # 5000006).

*ORC and Halo-tagged ORC*. ORC complex with a CBP-TEV tag on orc1 was purified from *S. cerevisiae* strain ySDORC, and ORC complex with a CBP-TEV-Halo tag on orc3 was purified from strain yTL158. Cells were seeded at a density of 2 × 10^7^ cells per ml in YP medium (1% yeast extract and 2% peptone) supplemented with 2% raffinose and grown at 30°C and 180 rpm till a density of 3–5 × 10^7^ cells/ml. Then cells were arrested in G1 by adding 100 ng/ml α-mating factor (Tebu-Bio #089AS-60221-5) for 3 h followed by the addition of 2% galactose for 3 h to induce the expression of ORC. Cells were harvested by centrifugation and washed with ORC lysis buffer (25 mM HEPES-KOH pH 7.6, 0.05% NP-40 substitute, 10% glycerol, 0.1 M KCl, and 1 mM DTT). After centrifugation, cells were suspended in ORC lysis buffer supplemented with protease inhibitors (cOmplete^TM^ EDTA-free Protease Inhibitors (Sigma-Aldrich #5056489001) and 0.3 mM phenylmethylsulfonyl fluoride (PMSF)) and dropped into liquid nitrogen. The frozen droplets were ground in a freezer mill, 6875 SPEX, for si cycles (run time 2 min and cool time 1 min with a rate of 15 cps), and the resulting powder was suspended in ORC lysis buffer supplemented with protease inhibitors. The lysate was cleared in a Beckman-Coulter ultracentrifuge (type Optima L90K with rotor TI45) for 1 h at 45,000 rpm and 4°C. The cleared lysate was supplemented with CaCl_2_ to a final concentration of 2 mM and with KCl to a final concentration of 0.3 M, and was incubated for 1 h at 4°C with washed Sepharose 4B Calmodulin beads (GE Healthcare #17-0529-01) in a spinning rotor. The beads were washed 20 times with 5 ml ORC-binding buffer (25 mM HEPES-KOH pH 7.6, 0.05% NP-40 substitute, 10% glycerol, 0.3 M KCl, 2 mM CaCl_2_, and 1 mM DTT), and the protein complex was eluted from the beads with ORC elution buffer (25 mM HEPES-KOH pH 7.6, 0.05% NP-40 substitute, 10% glycerol, 0.3 M KCl, 2 mM EDTA, 2 mM EGTA, and 1 mM DTT). ORC-containing fractions were pooled, concentrated in an Amicon Ultra-4 Ultracell 30 kDa centrifugal filter (Merck-Millipore #UFC803024), and applied to a Superose 6 increase 10/300 GL column (GE Healthcare #29-0915-96) equilibrated in ORC GF buffer (25 mM HEPES-KOH pH 7.6, 0.05% NP-40 substitute, 10% glycerol, 0.15 M KCl, and 1 mM DTT). Peak fractions were pooled and concentrated in an Amicon Ultra-4 Ultracell 30 kDa centrifugal filter (Merck-Millipore #UFC803024). Aliquots were snapfrozen and stored at −80°C. The protein concentration was determined with Bio-Rad Protein Assay Dye Reagent (Bio-rad # 5000006).

*Mcm2-7/Cdt1 and Halo-tagged Mcm2-7/Cdt1.* Mcm2-7/Cdt1 complex with a CBP-TEV tag on mcm3 was purified from *S. cerevisiae* strain yAM33, and Mcm2-7/Cdt1 complex with a CBP-TEV-Halo tag on mcm3 was purified from strain yTL001. Cells were grown and Mcm2-7/Cdt1 expression was induced as described for ORC. Cells were harvested by centrifugation, washed with Mcm lysis buffer (45 mM HEPES-KOH pH 7.6, 0.02% NP-40 substitute, 10% glycerol, 100 mM KOAc, 5 mM MgOAc, and 1 mM DTT). After centrifugation, cells were suspended in Mcm lysis buffer supplemented with protease inhibitors (cOmplete^TM^ EDTA-free Protease Inhibitors (Sigma-Aldrich #5056489001) and 0.3 mM PMSF) and dropped into liquid nitrogen. The frozen droplets were ground in a freezer mill (6875 SPEX) for six cycles (run time 2 min and cool time 1 min at a rate of 15 cps), and the resulting powder was suspended in Mcm lysis buffer supplemented with protease inhibitors. The lysate was cleared in a Beckman-Coulter ultracentrifuge (type Optima L90K with rotor TI45) for 1 h at 45,000 rpm and 4°C. The cleared lysate was supplemented with CaCl_2_ to a final concentration of 2 mM, and was then incubated for 1 h at 4°C with washed Sepharose 4B Calmodulin beads (GE Healthcare #17-0529-01) in a spinning rotor. The beads were washed 20 times with 5 ml Mcm binding buffer (45 mM HEPES-KOH pH 7.6, 0.02% NP-40 substitute, 10% glycerol, 100 mM KOAc, 5 mM MgOAc, 2 mM CaCl_2_, and 1 mM DTT), and the protein complex was eluted from the beads with Mcm elution buffer (45 mM HEPES-KOH pH 7.6, 0.02% NP-40 substitute, 10% glycerol, 100 mM KOAc, 5 mM MgOAc, 1 mM EDTA, 2 mM EGTA, and 1 mM DTT). Mcm2-7/Cdt1-containing fractions were pooled, concentrated in an Amicon Ultra-4 Ultracell 30 kDa centrifugal filter (Merck-Millipore #UFC803024), and applied to a Superose 6 increase 10/300 GL column (GE Healthcare #29-0915-96) equilibrated in Mcm GF buffer (45 mM HEPES-KOH pH 7.6, 0.02% NP-40 substitute, 10% glycerol, 100 mM KOAc, 5 mM MgOAc, and 1 mM DTT). Peak fractions were pooled and concentrated in an Amicon Ultra-4 Ultracell 30 kDa centrifugal filter (Merck-Millipore #UFC803024). Aliquots were snapfrozen and stored at −80°C. Protein concentration was determined with Bio-Rad Protein Assay Dye Reagent (Bio-rad # 5000006).

*dCas9-Halo.* Halo-tagged dCas9 protein expression^37^ was induced in BL21-CodonPlus(DE3)-RIL cells (Agilent #230245) transformed with pET302-6His-dCas9-halo (Addgen #72269) with 400 µM IPTG for 16 h at 16°C. Cells were harvested in dCas9 lysis buffer (50 mM Na_x_PO_4_ pH 7.0, 300 mM NaCl and protease inhibitors (cOmplete^TM^ EDTA-free Protease Inhibitors (Sigma-Aldrich #5056489001) plus 0.3 mM PMSF)) and sonicated in an Qsonica Q500 sonicator for 2 min with cycles of 5 s on and 5 s off and an amplitude of 40%. After centrifugation, dCas9-Halo protein was purified from the supernatant by incubating for 2 h at 4°C with Ni-NTA agarose (Qiagen #30210). The beads were washed 10 times with 5 ml dCas9 wash buffer I (50 mM Na_x_PO_4_ pH 7.0 and 300 mM NaCl) and three times with dCas9 wash buffer II (50 mM Na_x_PO4 pH 7.0, 300 mM NaCl, and 20 mM Imidazole pH 7.6), and dCas9-Halo was eluted from the agarose beads with dCas9 elution buffer (50 mM Na_x_PO_4_ pH 7.0, 300 mM NaCl, and 150 mM Imidazole pH 7.6). Subsequently, dCas9-Halo eluate was dialyzed twice for 1 h against dCas9-dialysis buffer (50 mM HEPES-KOH pH 7.6, 100 mM KCl, and 1 mM DTT) in a 10 kDa cutoff Slide-A-Lyzer Cassette (Thermo Scientific #66380) and applied to a Hi Trap SP HP column (GE Healthcare #17-1151-01) equilibrated with dCas9-dialysis buffer. The dCas9-Halo protein was eluted from the column with dialysis buffer with a KCl gradient ranging from 100 mM up to 1 M. The dCas9-Halo-containing fractions were pooled, concentrated in an Amicon Ultra-4 Ultracell 30 kDa centrifugal filter (Merck-Millipore #UFC803024), and applied to a Superdex 200 increase 10/300 GL column (GE Healthcare #28-9909-44) equilibrated in cas9 GF buffer (50 mM HEPES-KOH pH 7.6, 150 mM KCl, and 1 mM DTT). Peak fractions were pooled and concentrated in an Amicon Ultra-4 Ultracell 30 kDa centrifugal filter (Merck-Millipore #UFC803024). Aliquots were snapfrozen and stored at −80°C. The protein concentration was determined with Bio-Rad Protein Assay Dye Reagent (Bio-rad # 5000006).

#### Protein labeling

*Strains*. to create Halo-tagged mcm3, the StuI and XmaI restriction sites in plasmid pENTR4-halo-tag (Addgene #W876-1) were changed into a silent mutation following standard cloning techniques using primers TL-019-TL-020 and TL-023-TL-024. The sequence was verified by sequencing using primers TL-021-TL-022. Then the halo fragment was amplified from the mutated pENTR4-halo-tag by PCR with primers TL-025 and TL-026, which were extended with an XmaI site. This amplified halo fragment was digested with XmaI, gel-purified, and ligated into plasmid pRS306-CBP-TEV-mcm3-gal1-10 mcm2, which was digested with SgrAI and dephosphorylated with CIP, resulting in plasmid pRS306-CBP-TEV-mhalo-mcm3-gal1-10 mcm2. Proper integration of the Halo tag was confirmed by sequencing with primers (see Supplementary Table 1) TL-001, TL-002, TL-027, and TL-028. Yeast strain yTL001, which expresses MCM with a Halo-tagged mcm3, was created by linearizing plasmid pRS306-CBP-TEV-mhalo-mcm3-gal1-10 mcm2 with StuI and transforming it into yeast strain yJF21, which expresses Mcm4-7 and Cdt1 upon induction with galactose.

To create an ORC complex with a halo-tagged orc3, the CBP-TEV sites was removed from plasmid pRS306 Orc1-gal1-10-Orc2 through Gibson assembly (NEB #E2611L) using primers TL-441, TL-443, and TL-447. The sequence for the coding region of orc1 and orc2 was confirmed by sequencing using primers TL-084, TL-087, TL-119, and TL-136. Yeast strain yTL151, which expresses orc1, 2, 5, and 6 from a galactose-inducible promoter, was created by linearizing plasmid pRS306 Orcl-gal1-10-Orc2 v2 delta CBP-TEV with StuI and transforming it into yeast strain yTL070, which contains an inducible expression plasmid for orc5 and orc6.

Plasmid pRS303-CBP-TEV-halo-orc3-gal1-10 orc4 was generated by cloning the CBP-TEV-halo sequence from plasmid pRS306-CBP-TEV-halo-Pri1 -Gal1-10 Pri2 into plasmid pRS303-orc3-Gal1-10 orc4 through Gibson assembly (NEB #E2611L) using primers TL-446, TL-447, TL-472, and TL-473). The sequence of CBP-TEV-halo-orc3 and orc4 was verified by sequencing using primers TL-063, TL-064, TL-449, and TL-470. Yeast strain yTL158, which expresses ORC with a halo-tagged orc3, was created by linearizing plasmid pRS303-CBP-TEV-halo-orc3-Gal1-10 orc4 with NheI and transforming it into yeast strain yTL151, which contains inducible expression plasmids for orc1, orc2, orc5, and orc6.

*Labeling reactions*. Halo-tagged proteins were labeled with JF549-HaloTag and JF646-HaloTag ligands by incubating the proteins with a tenfold excess of dye on ice for 0.5–1 h in the presence of 1 mM ATP. The JF549-HaloTag and JF646-HaloTag ligands were a kind gift from Luke Davis (Janelia Farm). Free dye was removed by gel filtration (Superose 6 increase 10/300), and the labeling efficiency was determined to be at least 81% and 80% for JF549-ORC and JF646-MCM after estimating protein and fluorophore concentrations relative to known standards. Accordingly, we cannot exclude the possibility that ~20% of the observed single ORC or single MCM populations may have been partially labeled double ORC and double MCM hexamers.

#### DNA substrates

*Bulk loading assay*. 5.8 kbp circular bead-bound ARS1-containing pSK (+)-based plasmid^10^.

*Single-molecule experiments*. To generate a 21 kb plasmid containing insert sequence pGC203 (high-affinity origin), geneblock pGC203 (synthesized by Integrated DNA Technologies, see Supplementary Table 1) was amplified by PCR using primers TL-155 and TL-156, digested with AscI and cloned into MluI-digested and Antarctic-dephosphorylated plasmid pSupercos1-lambda 1,2^38^. The sequence and orientation were verified by sequencing using primers TL-037 and TL-157.

The 21 kb plasmid, containing insert sequence geneblock pGC218 (high-affinity origin with point mutant, synthesized by Integrated DNA Technologies, see Supplementary Table 1), was amplified by PCR using primers TL-169 and TL-156, digested with AscI, and cloned into MluI-digested and Antartic-dephosphorylated plasmid pSupercos1-lambda 1,2. The sequence and orientation were verified by sequencing using primers TL-037 and TL-157.

To generate a 21 kb fragment of plasmid pSupercos1-lambda 1,2, containing either insert sequence pGC203 or pGC218, the plasmid was linearized via digestion with AflII. The four-nt overhangs were biotinylated by incorporation of biotin-labeled dATP, dUTP, and native dGTP, dCTP, by Klenow fragment exo-, resulting in two biotins at each end of the DNA^39^.

### Bulk assays and single-molecule experiments

#### MCM recruitment and loading reactions in bulk

Loading assays were carried out as follows: 50 nM ORC (or JF549-ORC), 50 nM Cdc6, and 100 nM Mcm2-7/Cdt1 (or JF646-Mcm2-7/Cdt1) were incubated with 300 ng DNA substrate coupled to magnetic beads for 30 min at 30 °C with mixing at 1250 RPM (tubes) in 40 μl reaction buffer (25 mM HEPES-KOH pH 7.6, 10 mM MgOAc, 100 mM KOAc, 0.02% NP-40, 5% glycerol, 1 mM DTT, 5 mM ATP or ATPγS). Beads were then washed either with high-salt wash buffer (45 mM HEPES-KOH pH 7.6, 5 mM MgOAc, 0.5 M NaCl, 0.02% NP-40, 10% glycerol, 1 mM EDTA, 1 mM EGTA) followed by low salt wash buffer (45 mM HEPES-KOH pH 7.6, 5 mM MgOAc, 0.3 M KOAc, 0.02% NP-40, 10% glycerol, 1 mM EDTA, 1 mM EGTA), or only treated with low salt wash buffer. Finally, beads were resuspended in 10 μl elution buffer (45 mM HEPES-KOH pH 7.6, 5 mM MgOAc, 0.3 M KOAc, 10% glycerol, 2 mM CaCl_2_), and DNA-bound proteins were released by MNase treatment (2 min 30° with 700 units of MNase NEB # M0247S) and analyzed by gel electrophoresis^14^.

### Single-molecule instrumentation and visualization

Visualization of DNA–protein binding at the single-molecule level was performed using a hybrid instrument that combines optical tweezers and confocal microscopy (Q-Trap, LUMICKS). The instrument makes use of a microfluidic chip with five inlets and one outlet, arranged such that three of the five reaction buffers are injected from the left and the other two are introduced orthogonally and can be used as protein reservoirs or buffer exchange locations in a temperature-controlled environment. Syringes and tubing connected to the chip were passivated, together with the chip itself, with 1 mg/mL bovine serum albumin (BSA) followed by 0.5% Pluronic F-127 (Sigma), each incubated for at least 30 min. Next, 1 pM of the biotinylated DNA, containing either a functional origin of replication or a mutated origin, was injected into one of the five laminar-flow-separated channels. Individual DNA molecules were trapped between two 1.76-μm streptavidin-coated polystyrene beads (Spherotech) initially injected into a separate channel.

In all measurements, the stiffnesses of both optical traps were set to 0.3 pN/nm^39–41^. The tethering of individual DNA molecules was verified by analysis of the force–extension curve obtained for each DNA molecule^42^ that was used for protein visualization. During fluorescence measurements, the DNA was held at a constant tension of 2 pN and the flow was turned off, unless otherwise specified. The JF549 and JF646 dyes were illuminated with two laser lines at 561 nm (7 µW) and 638 nm (7 µW), respectively, and the fluorescence from the dyes was detected on a single photon counting detector. Two-dimensional confocal scans were performed over an area of 140 × 40 pixels, which encompasses the DNA held at a force of 2 pN and the edges of the beads, or 200 × 40 pixels when including full beads in the image. The pixel size was set to 50 × 50 nm^2^, and the illumination time per pixel was set to 0.05 ms.

### Protein concentrations and buffers in single-molecule experiments

Incubation and visualization of DNA–protein interactions in the flow cell were performed at 30°C. ORC binding was conducted in reaction buffer (RB) containing 25 mM HEPES-KOH (pH 7.6), 100 mM potassium glutamate, 10 mM magnesium acetate, 100 μg/mL BSA, 1 mM DTT, 0.01% NP-40-S, 10% glycerol, 5 mM ATP, or ATPγS, with or without 10 nM Cdc6, and 5 nM ORC (unless otherwise stated). MCM recruitment was conducted in RB containing 5 mM ATPγS, with 10 nM Cdc6, 5 nM ORC, and 100 nM Mcm2-7/Cdt1. MCM loading reactions were done using ATP and maintaining the other components. Reactions were incubated for the indicated period of time (2–16 min), at which point DNA tethers were immersed either in the same buffer free of protein or in washing buffer (25 mM HEPES-KOH (pH 7.6), 500 mM sodium chloride, 100 μg/mL BSA, 1 mM DTT, 0.01% NP-40-S, 10% glycerol). To reduce the rate of photobleaching, we add 2 mM 1,3,5,7 cyclooctatetraene, 2 mM 4-nitrobenzylalchohol and 2 mM TROLOX.

Preparation of DNA–protein complexes in bulk for subsequent visualization in the flow cell was done as follows. In all, 5 nM ORC was incubated with 1 nM 21.2 kbp biotinylated DNA at 30°C while mixing at 1250 rpm in RB with 5 mM ATP (or ATPγS). After 5 min, 10 nM Cdc6 was added to the reaction and incubated for a further 5 min. In all, 100 nM MCM-Cdt1 was added, bringing the total reaction volume to 20 μl. After a 30 min incubation, samples were held on ice until being diluted 1000× in RB and injected into the microfluidic chip. ORC-only DNA complexes were assembled in the same conditions while omitting the other proteins.

### Data analysis

#### Particle localization in 2D scans

We used the open-source single-particle tracking ImageJ plugin TrackMatev5.2.0^43^ to identify and track particles bound to DNA. As detailed in ref. ^43^, TrackMate identifies regions of enhanced fluorescence, which are segmented and tracked from frame to frame. For each focus, TrackMate produces a set of coordinates (x, y, t) per frame, as well as maximum, minimum, and cumulative intensities within a defined radius of the center coordinate. We performed subpixel localization on foci identified in a median-filtered image stack, defining the intensity per frame as the cumulative intensity of a circular region with a radius of four pixels centered on the fitted (x,y) coordinates. We algorithmically allowed the gap between foci to close, but did not allow identified foci to either merge or split. For all the subsequent analyses, the intensity, position and time coordinates for individual tracked foci are exported from TrackMatev5.2.0 to Python 3.8 (with analysis libraries numpy 1.19.0, scipy 1.5.0, scikit-image 0.17.2, scikit-learn 0.23.1, and pandas 1.05) as XML files.

#### Localization distributions and offset correction

The HtH origin is located 6.7 kbp from one end of the 21.2 kbp DNA tether, but the tether can be oriented in either of two ways between the beads. To determine the occupancy of the original sequence, we used a radial localization histogram that plots the particle positions as a function of distance from the center of the DNA. We performed this mapping in two steps: first, subtraction of the horizontal offset in DNA position owing to the beads at the edges of the image, and averaging of histogram bins at equal distances from the center on opposite sides.

To determine the offset, which is caused by both the variability of the microsphere diameter (±0.2 µm) and slight variations in the position of the scan region relative to the tracked microspheres, we first plotted the raw localization distribution by taking the mean of the first five position measurements of each particle on each DNA molecule in the data set. In data sets with a symmetrical two-peaked localization distribution, corresponding to an enriched population at the origin (right of center on 50% of DNA molecules, and left of center on the other 50% of DNA molecules), we selected the offset that maximizes the autocorrelation of the two peaks. In data sets for which there is no clear bimodal distribution, we used microsphere position metadata exported directly from the instrument. Since the position value exported by the system depends on a user-defined tracking region, there is a possibility of systematic bias, hence our preference for the autocorrelation method when possible. However, in virtually all cases the offset needed to align the 2D scans, including those taken months apart, was in a range of 0.4 ± 0.3 µm. In the data sets collected on a mutated HtH origin in Fig. 1 and Supplementary Fig. 5, no metadata was exported, so we performed a manual offset correction using offset value 0.4 µm.

Once the offset has been applied, the independent DNA images are aligned, and the localization histogram is constructed by averaging the occupancies of bins on opposite sides of the center. We exclude localizations >10.6 kb from the center of the DNA, which correspond to false positives from fluorescent proteins adhered to the beads. These edge effects account for the majority of bright “noise” foci, but we also exclude foci elsewhere on the DNA with an intensity >10x the single-fluorophore intensity, which represents protein aggregation.

In our analysis, we consider a focus to be localized at the origin if it is within our localization error, 0.2 µm or 0.67 kb, of the HtH origin site. It is worth noting that we chose a slightly smaller histogram bin size, 0.59 kb, to ensure the HtH origin site was centered in a bin for maximum visual clarity.

#### Bleaching steps

We used the mathematical apparatus behind the Python sklearn decision tree regressor to fit bleaching steps to the raw intensity data, yielding lifetimes and stoichiometries (by step counting). In the background, this algorithm fits a stepwise function to an input data set by minimizing the mean squared error. After this fit, we combined steps that are too small by “pruning”. Finally, we corrected for large steps by dividing the detected step size by the average step size, and, if the quotient was larger than one, we treated the large step as multiple proteins dissociating or multiple fluorophores bleaching at once. To remove outliers, we eliminated any traces with a step count larger than 10 from our data sets.

For this fit, we needed to set a minimum step size and an average step size. To that end, we made bleaching step fits on dCas9 calibration data using an initial minimum step size estimate, yielding step size distributions. We then set the updated minimum step size to less than or equal to the mean minus two standard deviations of the distribution, in order to include at least 95% of steps. This process was repeated until the minimum step size value converged, yielding an average step size.

#### Fitting MSD curves to obtain diffusion constants

We used mean squared displacement (MSD) analysis of individual tracked foci as a function of the delay time between frames. The diffusion constant is the slope of a linear (unconfined) MSD curve. Using published analysis to determine the appropriate range of delay times for fitting^44^, we calculated the mean diffusion constant *D* for each tracked particle. We then fit the distribution of log(*D*) to a Gaussian Mixture Model to distinguish between kinetically distinct populations. In almost all of the environments studied, a two-state model was statistically preferred over a single state using the Bayesian Information Criterion. We identified these states as a static population (*D*~0.01 kbp^2^ s^−1^) and a diffusive population (*D*~0.1–1 kbp^2^ s^−1^).

We calculated the mean, variance, and standard error (*m*, var, SEM) of the *i*^th^ diffusion coefficient distribution from the fitted log-normal parameters *µ*_*i*_ and *σ*_*i*_ according to

$$m_i = \frac{1}{{\log (e)}}e^{\ln (10)\mu _i + (\ln \left( {10} \right)\sigma _i)^2/2}$$

$${\mathrm{var}}_i = \frac{1}{{\log (e)}}e^{2\ln (10)\mu _i + (\ln \left( {10} \right)\sigma _i)^2/2}\left( {e^{(\ln \left( {10} \right)\sigma _i)^2} - 1} \right)$$

$${\mathrm{SEM}}_i = \sqrt {{\mathrm{var}}_i/{\mathrm{N}}_i}$$

where the factors of log(*e*) and ln(10) account for our use of base 10, and N_*i*_ is the number of values in the data set times the area of the *i*^th^ fitted peak.

It is worth noting that the dependence of *m*_*i*_ on both *µ*_*i*_ and *σ*_*i*_ yields mean diffusion constants larger than might be inferred by simply computing $10^{\mu _{\mathrm{i}}}$.

### Figure schematics

Figure schematics were generated using BioRender.com (Standard Academic License).

### Reporting summary

Further information on research design is available in the Nature Research Reporting Summary linked to this article.

## Supplementary information

Supplementary Information

Description of Additional Supplementary Files

Supplementary Movie 1

Supplementary Movie 2

Supplementary Movie 3

Supplementary Movie 4

Supplementary Movie 5

Reporting Summary

## Data Availability

The data that support the findings of this study are available from the authors upon reasonable request. Source data are provided with this paper.

## Source data

Source Data

## Code availability

The analysis of the acquired data was performed using custom-written scripts in Python 3.8, which are available upon request.

## References

[1] Bell SP, Labib K (2016). Chromosome duplication in Saccharomyces cerevisiae. Genetics.

[2] Bell SP, Stillman B (1992). ATP-dependent recognition of eukaryotic origins of DNA replication by a multiprotein complex. Nature.

[3] Liachko I, Youngblood RA, Keich U, Dunham MJ (2013). High-resolution mapping, characterization, and optimization of autonomously replicating sequences in yeast. Genome Res..

[4] Hoggard T, Shor E, Müller CA, Nieduszynski CA, Fox CA (2013). A link between ORC-origin binding mechanisms and origin activation time revealed in budding yeast. PLoS Genet..

[5] Remus D (2009). Concerted loading of Mcm2-7 double hexamers around DNA during DNA replication origin licensing. Cell.

[6] Evrin C (2009). A double-hexameric MCM2-7 complex is loaded onto origin DNA during licensing of eukaryotic DNA replication. Proc. Natl. Acad. Sci. USA.

[7] Abid Ali F (2017). Cryo-EM structure of a licensed DNA replication origin. Nat. Commun..

[8] Noguchi Y (2017). Cryo-EM structure of Mcm2-7 double hexamer on DNA suggests a lagging-strand DNA extrusion model. Proc. Natl Acad. Sci. USA.

[9] Douglas ME, Ali FA, Costa A, Diffley JFX (2018). The mechanism of eukaryotic CMG helicase activation. Nature.

[10] Yeeles JTP, Deegan TD, Janska A, Early A, Diffley JFX (2015). Regulated eukaryotic DNA replication origin firing with purified proteins. Nature.

[11] Rhind N, Gilbert DM (2013). DNA replication timing. Cold Spring Harb. Perspect. Biol..

[12] Yekezare M, Gó mez-González B, Diffley JFX, Controlling DNA (2013). replication origins in response to DNA damage - inhibit globally, activate locally. J. Cell Sci..

[13] Sedlackova H (2020). Equilibrium between nascent and parental MCM proteins protects replicating genomes. Nature.

[14] Frigola J, Remus D, Mehanna A, Diffley JFX (2013). ATPase-dependent quality control of DNA replication origin licensing. Nature.

[15] Fernández-Cid A (2013). An ORC/Cdc6/MCM2-7 complex is formed in a multistep reaction to serve as a platform for MCM double-hexamer assembly. Mol. Cell.

[16] Ticau S (2017). Mechanism and timing of Mcm2-7 ring closure during DNA replication origin licensing. Nat. Struct. Mol. Biol..

[17] Zhai Y (2017). Open-ringed structure of the Cdt1-Mcm2-7 complex as a precursor of the MCM double hexamer. Nat. Struct. Mol. Biol..

[18] Frigola J (2017). Cdt1 stabilizes an open MCM ring for helicase loading. Nat. Commun..

[19] Li N (2015). Structure of the eukaryotic MCM complex at 3.8 Å. Nature.

[20] Yuan Z (2017). Structural basis of Mcm2-7 replicative helicase loading by ORC-Cdc6 and Cdt1. Nat. Struct. Mol. Biol..

[21] Li N (2018). Structure of the origin recognition complex bound to DNA replication origin. Nature.

[22] Ticau S, Friedman LJ, Ivica NA, Gelles J, Bell SP (2015). Single-molecule studies of origin licensing reveal mechanisms ensuring bidirectional helicase loading. Cell.

[23] Coster G, Diffley JFX (2017). Bidirectional eukaryotic DNA replication is established by quasi-symmetrical helicase loading. Science.

[24] Miller TCR, Locke J, Greiwe JF, Diffley JFX, Costa A (2019). Mechanism of head-to-head MCM double-hexamer formation revealed by cryo-EM. Nature.

[25] Warner MD, Azmi IF, Kang S, Zhao Y, Bell SP (2017). Replication origin-flanking roadblocks reveal origin-licensing dynamics and altered sequence dependence. J. Biol. Chem..

[26] Gros J (2015). Post-licensing specification of eukaryotic replication origins by facilitated Mcm2-7 sliding along DNA. Mol. Cell.

[27] Prioleau MN, MacAlpine DM (2016). DNA replication origins—where do we begin?. Genes Dev..

[28] Donovan S, Harwood J, Drury LS, Diffley JFX (1997). Cdc6p-dependent loading of Mcm proteins onto pre-replicative chromatin in budding yeast. Proc. Natl Acad. Sci. USA.

[29] Bowers JL, Randell JCW, Chen S, Bell SP (2004). ATP hydrolysis by ORC catalyzes reiterative Mcm2-7 assembly at a defined origin of replication. Mol. Cell.

[30] Speck C, Stillman B (2007). Cdc6 ATPase activity regulates ORC·Cdc6 stability and the selection of specific DNA sequences as origins of DNA replication. J. Biol. Chem..

[31] Kochaniak AB (2009). Proliferating cell nuclear antigen uses two distinct modes to move along DNA. J. Biol. Chem..

[32] Schmidt JM, Bleichert F (2020). Structural mechanism for replication origin binding and remodeling by a metazoan origin recognition complex and its co-loader Cdc6. Nat. Commun..

[33] Zaugg JB, Luscombe NM (2012). A genomic model of condition-specific nucleosome behavior explains transcriptional activity in yeast. Genome Res..

[34] Ibarra A, Schwob E, Méndez J, Excess MCM (2008). proteins protect human cells from replicative stress by licensing backup origins of replication. Proc. Natl Acad. Sci. USA.

[35] Alver RC, Chadha GS, Blow JJ (2014). The contribution of dormant origins to genome stability: from cell biology to human genetics. DNA Repair (Amst.).

[36] Dequeker, B. J. H. et al. MCM complexes are barriers that restrict cohesin-mediated loop extrusion. *bioRxiv* (2020) 10.1101/2020.10.15.340356. 10.1038/s41586-022-04730-0 PMC9159944 35585235

[37] Deng W, Shi X, Tjian R, Lionnet T, Singer RH (2015). CASFISH: CRISPR/Cas9-mediated in situ labeling of genomic loci in fixed cells. Proc. Natl Acad. Sci. USA.

[38] Van Loenhout MTJ, De Grunt MV, Dekker C (2012). Dynamics of DNA supercoils. Science.

[39] Forget AL, Dombrowski CC, Amitani I, Kowalczykowski SC (2013). Exploring protein-DNA interactions in 3D using in situ construction, manipulation and visualization of individual DNA dumbbells with optical traps, microfluidics and fluorescence microscopy. Nat. Protoc..

[40] Candelli A (2014). Visualization and quantification of nascent RAD51 filament formation at single-monomer resolution. Proc. Natl. Acad. Sci. USA.

[41] Wasserman MR, Schauer GD, O’Donnell ME, Liu S (2019). Replication fork activation is enabled by a single-stranded DNA gate in CMG helicase. Cell.

[42] Bustamante C, Marko JF, Siggia ED, Smith S (1994). Entropic elasticity of λ-phage DNA. Science.

[43] Tinevez JY (2017). TrackMate: an open and extensible platform for single-particle tracking. Methods.

[44] Michalet X (2010). Mean square displacement analysis of single-particle trajectories with localization error: Brownian motion in an isotropic medium. Phys. Rev. E Stat. Nonlin. Soft Matter Phys..

