# Supplementary material for: DNA replication origins retain mobile licensing proteins
Source: Nat Commun. 2021 Mar 26;12:1908. doi: 10.1038/s41467-021-22216-x (PMC7998030; doi:10.1038/s41467-021-22216-x)
Supplement: Supplementary file 1 — Supplementary Information [file 41467_2021_22216_MOESM1_ESM.pdf]

Supplementary Information for:

DNA replication origins retain mobile licensing proteins

Humberto Sánchez[1], Kaley McCluskey[1], Theo van Laar[1], Edo van Veen[1], Filip M. Asscher[1], Belén Solano[1], John F.X. Diffley*[2], and Nynke H. Dekker*[1]

[1]Department of Bionanoscience, Kavli Institute of Nanoscience, Delft University of Technology, van der Maasweg 9, 2629 HZ Delft, The Netherlands

[2]Chromosome Replication Laboratory, Francis Crick Institute, London, United Kingdom

*Correspondence to: John.Diffley@crick.ac.uk; n.h.dekker@tudelft.nl

Supplementary Figures

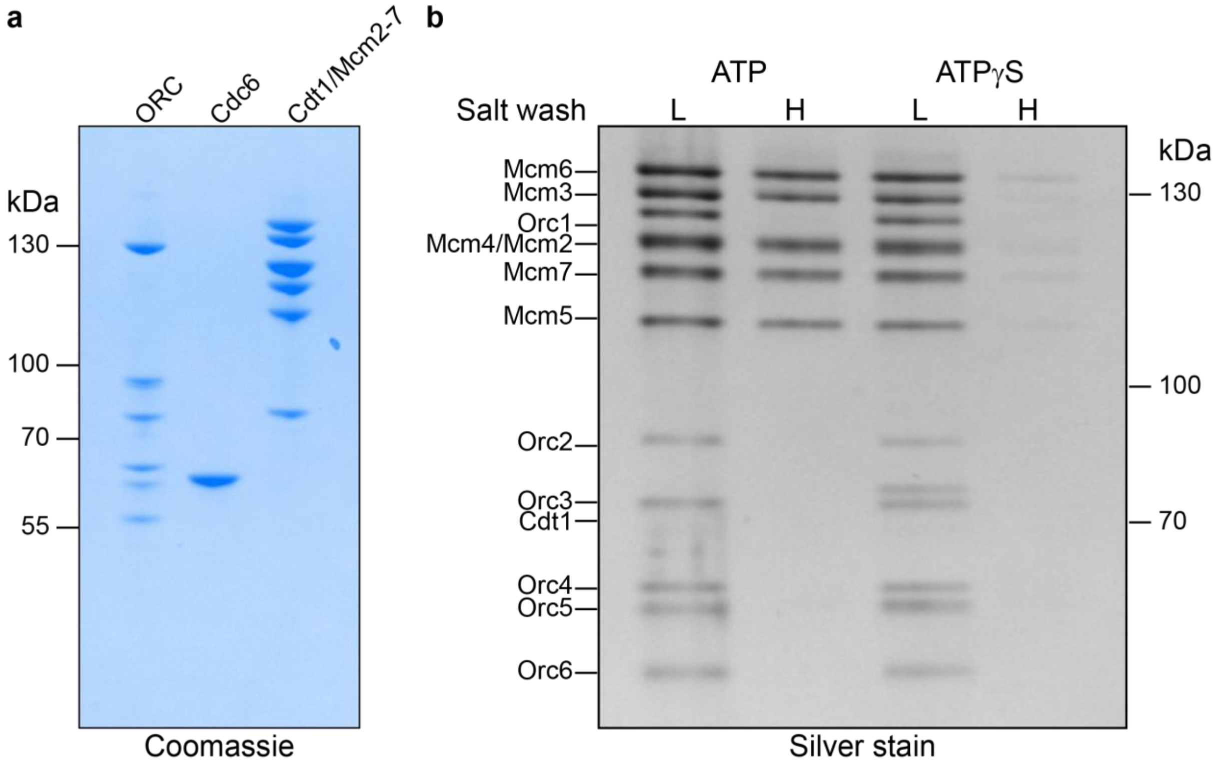

Supplementary Figure 1. Purification of unlabeled proteins and loading assay.
(a) SDS-PAGE gel of the unlabeled loading proteins ORC, Cdc6, and Mcm2-7/Cdt1. (b) Loading assay performed on bead-tethered DNA in bulk using unlabeled proteins. Following a reaction performed in the presence of either ATP or ATPγS, either a low (L) or a high-salt (H) wash is performed. DNA is removed from the beads by MNase treatment, and the presence of DNA-bound proteins is examined using SDS-PAGE followed by silver staining. Mcm2-7 proteins are retained on the DNA following a high-salt wash only if the loading reaction was performed in the presence of ATP. This confirms recruitment of Mcm2-7 in the presence of ATPγS, but loading of Mcm2-7 only in the presence of ATP. Experiments were reproduced at least three times.

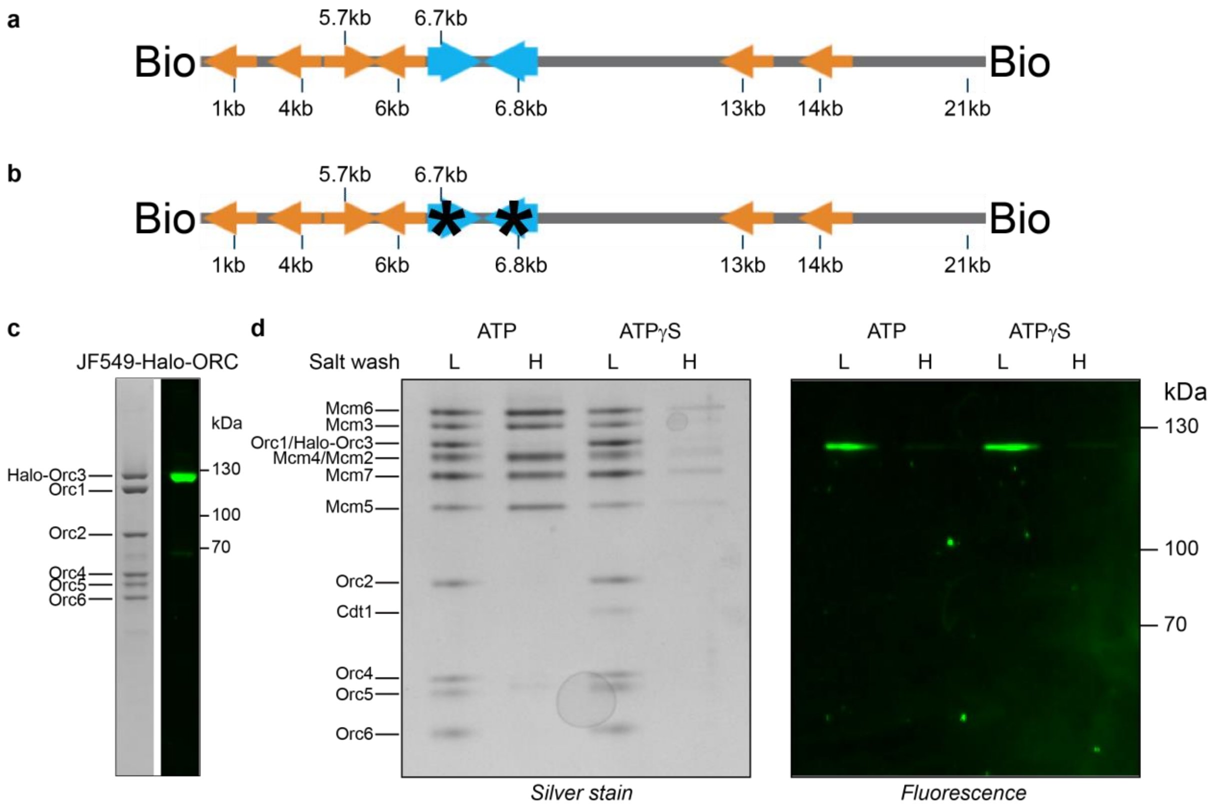

Supplementary Figure 2. DNA molecules for single-molecule experiments, purification of labeled ORC, and loading assay with labeled ORC.

(a) Schematic of the principal DNA construct (21.2 kbp) tagged with multiple biotins at both extremities. The origin, containing high-affinity ORC binding sites (blue arrows), is located 6.7 kbp from one extremity. Potential ORC binding sites are also indicated (orange arrows), assigned based on a position weight matrix covering the entire extended ACS as previously described [1]. (b) The same DNA construct as in panel (a), but containing a mutated origin (mHtH) in which five nucleotides of the high affinity ORC binding sites (blue arrows) have been substituted to render the origin inactive [2]. The other potential ORC binding sites (orange arrows), have not been modified. (c) The ORC complex, including a HaloTag on the Orc3 subunit labeled with a single JF549 dye (labeling efficiency estimated at 80%), examined on SDS-PAGE (left) with Coomassie staining and (right) upon laser excitation. Uncropped gel images are available as Source_data. (d) Loading assay performed as described in Supplementary Fig. 1, in the presence of JF549-ORC. Products are run on SDS-PAGE gel and imaged using (left) silver staining and (right) fluorescence. Experiments were reproduced at least three times.

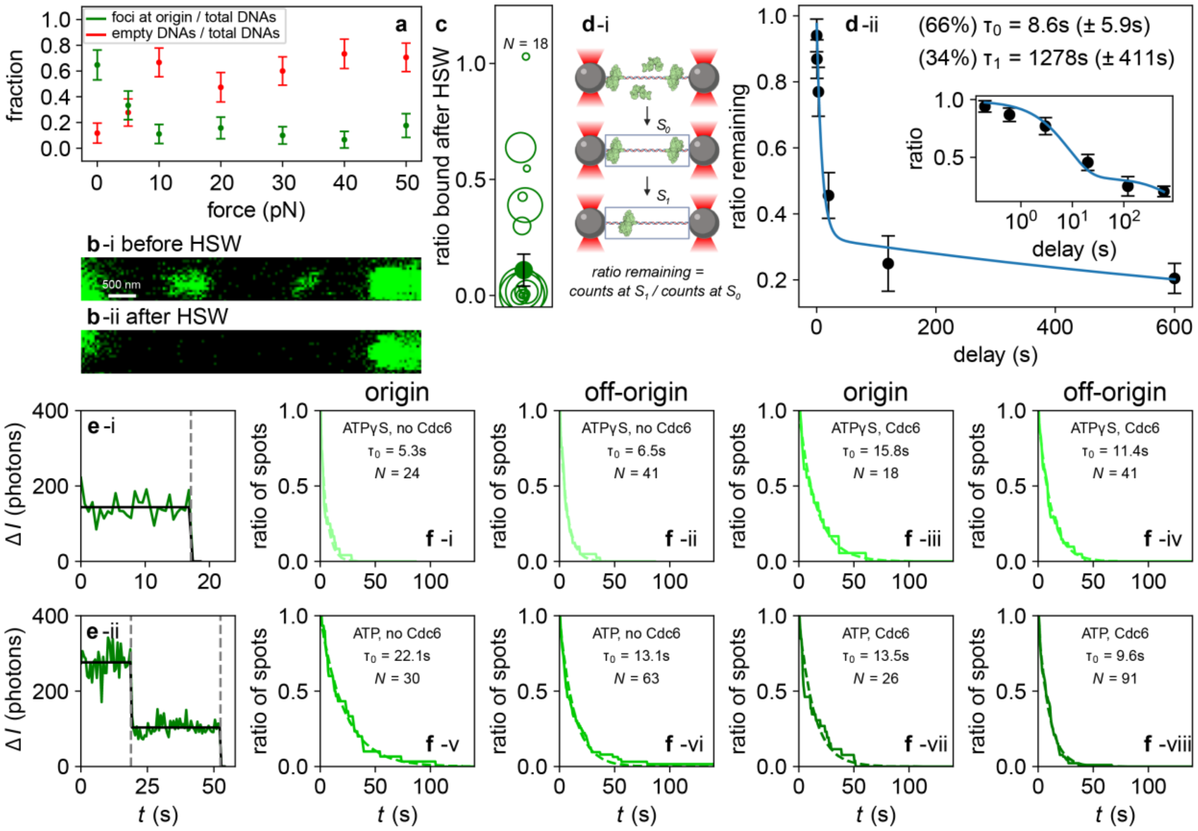

Supplementary Figure 3. Stability of labeled ORC binding to DNA.

(a) The effect of tension on the initial ORC-DNA interaction. Shown are the number of empty DNA molecules (red) and ORC foci on the origin (green), both normalized by the total number of DNA molecules tested, visualized after incubation for 2 min at the forces indicated (filled circles and error bars indicate mean ± S.D.; N_{scans} = 106; N_{foci} = 93). (b) (i) Example image (n=18, analysed in panel c) showing JF549-ORC bound to a single, trapped 21.2 kbp DNA following 2 min incubation at near-zero force (similar to Figure 1a). (ii) Following a high-salt wash of this DNA molecule, no remaining fluorescence from JF549-ORC is observed. (c) Ratio of the fluorescence emitted by all JF549-ORC molecules found bound to DNA before and after a high-salt wash. The circles indicate individual scans; their sizes indicate the total photon intensity in the scan frames. The filled circle represents the average ratio over all scans, and error bars indicate S.D. (d) (i) Experimental workflow to measure the fraction of DNA-bound ORC over time in the absence of photobleaching. The total fluorescence emitted by all JF549-ORC molecules bound to DNA following a 2-min incubation of JF549-ORC and DNA in the flow cell is measured nearly immediately (at time S_0) following the incubation. Following a delay time in the dark S_1, the total fluorescence emitted by all JF549-ORC molecules bound to DNA is remeasured. (ii) Ratio of the total fluorescence measured at delay time S_1 relative to time S_0. The data (N = 49; represented as mean ± S.D.) is best fit to a biexponential function, yielding two time constants, t_0 = 8.6 ± 5.9 s and t_1 = 1278 ± 411 s. The inset shows the same data plotted on a log scale. (e) Bleaching steps observed for JF549-ORC bound to DNA. The step-fitting procedure that results in the fitted steps (solid black lines) is described in the Methods. (f) Lifetime measurements of individual ORC molecules initially detected "on origin" (within 0.2 µm of the HtH site) and "off-origin" (more than 0.2 µm away). 0.2 µm corresponds to our resolution limit for PSF localization. Mean lifetimes τ_0 are fit to the indicated number of data points for: (i, ii) incubation of ORC in ATPγS; (iii, iv) incubation of ORC and Cdc6 in ATPγS; (v, vi) incubation of ORC in ATP; (vii, viii) incubation of ORC and Cdc6 in ATP. As one can observe, the fitted lifetimes are relatively short compared to the bleaching time of an individual JF549 dye (Supplementary Fig. 5b-iv).

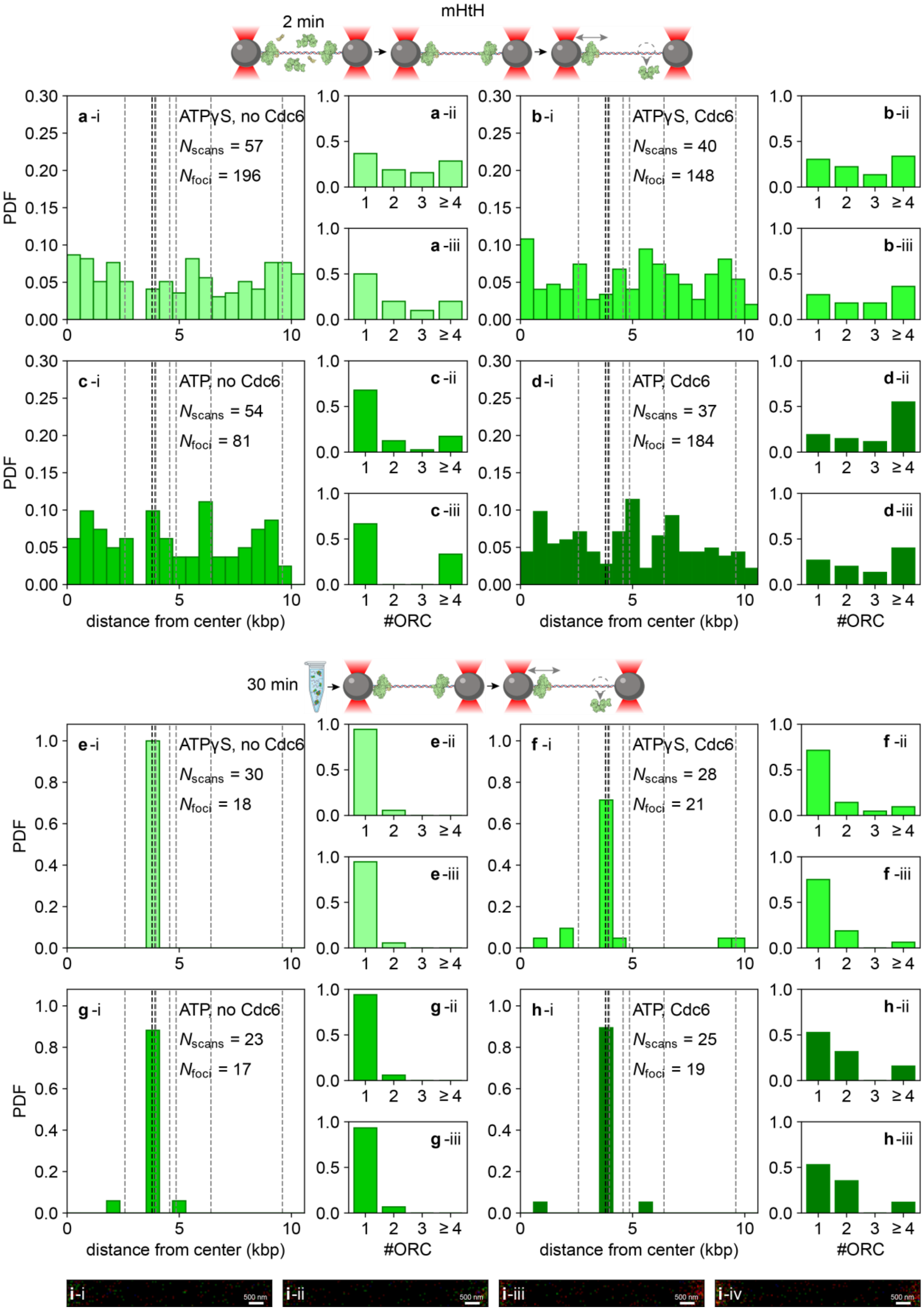
2 min
mHtH
a-i
ATPγS, no Cdc6
N_{scans} = 57
N_{foci} = 196
a-ii
a-iii
b-i
ATPγS, Cdc6
N_{scans} = 40
N_{foci} = 148
b-ii
b-iii
c-i
ATP, no Cdc6
N_{scans} = 54
N_{foci} = 81
c-ii
c-iii
d-i
ATP, Cdc6
N_{scans} = 37
N_{foci} = 184
d-ii
d-iii
PDF
distance from center (kbp)
#ORC
30 min
e-i
ATPγS, no Cdc6
N_{scans} = 30
N_{foci} = 18
e-ii
e-iii
f-i
ATPγS, Cdc6
N_{scans} = 28
N_{foci} = 21
f-ii
f-iii
g-i
ATP, no Cdc6
N_{scans} = 23
N_{foci} = 17
g-ii
g-iii
h-i
ATP, Cdc6
N_{scans} = 25
N_{foci} = 19
h-ii
h-iii
i-i
i-ii
i-iii
i-iv
500 nm

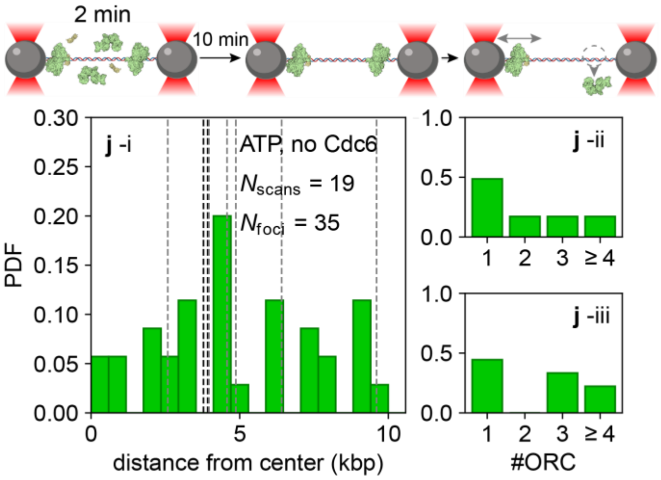

Supplementary Figure 4. The spatial distribution of ORC on DNA including a mutated origin, on DNA following a 30 min incubation in bulk, and following a 10 min delay between incubation and imaging in the flow cell.

(a-d) The loading of ORC onto long molecules of DNA with a mutated origin. (i) Histograms of the spatial distribution of fluorescent ORC foci, imaged over 5 s following a 2-min incubation of a single 21.2 kbp DNA containing the mHtH origin (described in Supplementary Fig. 2) with JF549-ORC. Specific conditions are: (a) incubation of ORC in ATPγS; (b) incubation of ORC and Cdc6 in ATPγS; (c) incubation of ORC in ATP; (d) incubation of ORC and Cdc6 in ATP. (ii, iii) Distributions of the stoichiometry of these foci, both (ii) overall and (iii) within 0.2 µm, or 0.67 kb, of the mHtH origin. (e)-(h) (i) Histograms of the spatial distribution of fluorescent ORC foci, imaged over 5 s following a 30-min bulk incubation of JF549-ORC and 21.2 kbp DNA. The DNA was then introduced into the experimental chamber, and the proteins were visualized. Specific conditions are: (e) incubation of ORC in ATPγS; (f) incubation of ORC and Cdc6 in ATPγS; (g) incubation of ORC in ATP; (h) incubation of ORC and Cdc6 in ATP. (ii, iii) Distributions of the stoichiometry of these foci, both (ii) overall and (iii) within 0.2 µm, or 0.67 kb, of the HtH origin. Panel (i): Experimental protocol as in panels (e)-(h), but with the DNA containing the mHtH origin. Representative images demonstrate the lack of any observable ORC binding under these conditions: (i) incubation of ORC in ATPγS (50 DNA visualized, $N_{green\ foci}$=1); (ii) incubation of ORC and Cdc6 in ATPγS (50 DNA visualized, $N_{green\ foci}$ =1); (iii) incubation of ORC in ATP (40 DNA visualized, $N_{green\ foci}$ =0); (iv) incubation of ORC and Cdc6 in ATP (30 DNA visualized, $N_{green\ foci}$ =0). (j) Impact of a period in protein-free buffer in the flow cell on fluorescent ORC foci. (i) Histogram of the spatial distribution of DNA-bound JF549-ORC foci imaged over 5 s following a 2 min-long incubation of a single 21.2 kbp DNA with JF549-ORC and subsequent placement in protein-free buffer in the flow cell for 10 min. (ii, iii) Distributions of the stoichiometry of these foci, both (ii) overall and (iii) within 0.2 µm, or 0.67 kb, of the HtH origin.

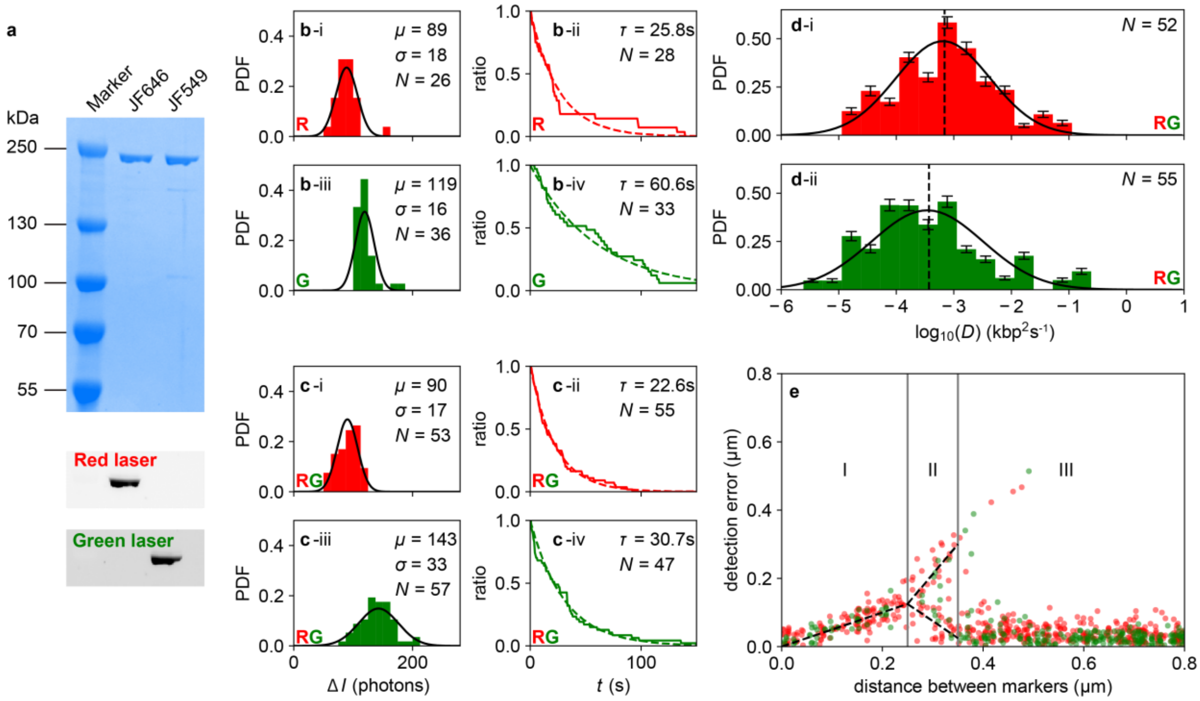

Supplementary Figure 5. Fluorescently labeled dCas9 proteins as standards for determination of stoichiometry and localization accuracy.

(a) SDS-PAGE gels of HaloTagged dCas9 labeled with JF646 and JF549. Shown are (top) Coomassie and (bottom) fluorescence images for dCas9-JF646 and dCas9-JF549, respectively. Uncropped gel images are available as Source_data. (b) Distributions of intensity changes and decay times associated with photobleaching for (i, ii) dCas9-JF646 and (iii, iv) dCas9-JF549 upon single-color illumination with lasers at 638 nm and 561 nm, respectively (represented in-panel by the letters R and G). (c) Distributions of intensity changes and decay times associated with photobleaching for (i, ii) dCas9-JF646 and (iii, iv) dCas9-JF549 upon dual-color illumination with lasers at 561 nm and 638 nm (represented in-panel by the letters RG). (d) Diffusion coefficient distribution of (i) dCas9-JF646 and (ii) dCas9-JF549 upon dual-color illumination with lasers at 638 nm and 561 nm. A lognormal fit places the mean diffusion coefficients at 0.010 ± 0.006 kbp^2 s^{-1} for dCas9-JF646 and 0.01 ± 0.01 kbp^2 s^{-1} for dCas9-JF549 (reported as mean ± SEM). (e) The accuracy with which a tagged protein can be located on the tethered DNA, assessed using simulated foci. The foci are generated using the PSFs from dCas9-JF549 and dCas9-JF646 upon dual-color illumination with lasers at 561 nm and 638 nm, with shot noise added. TrackMate is then used to detect these foci. Shown are the deviations from the true focus locations as a function of the distance between generated foci, with dashed lines as guides for the eyes. In region I, TrackMate puts a detection marker between of the two foci. In region II, TrackMate puts a detection marker on one of the foci, but misses the other, as there would be considerable overlap between the markers. Hence, the scatter plot splits in two. In region III, TrackMate can resolve both individual foci.

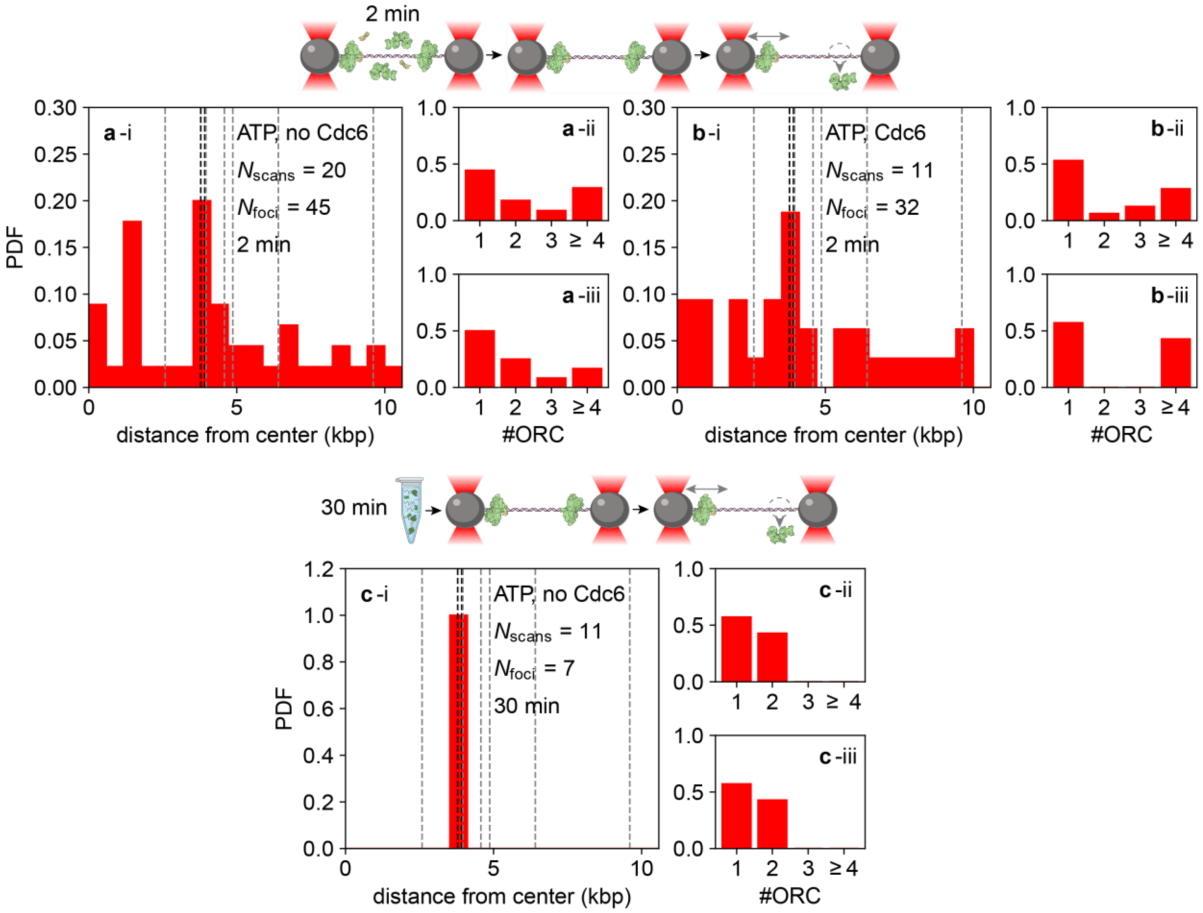

Supplementary Figure 6. The spatial distribution of JF646-ORC under different biochemical conditions, and following a 30 min incubation in bulk.
(a,b) (i) Histograms of the spatial distribution of red-fluorescent ORC foci following a 2-min incubation of a single 21.2 kbp DNA with 5 nM JF646-ORC. Specific conditions are: (a) incubation of ORC in ATP; (b) incubation of ORC and Cdc6 in ATP. (c) (i) Histogram of the spatial distribution of fluorescent ORC foci, following a 30-min bulk incubation of JF646-ORC with 21.2 kbp DNA. The DNA was then introduced into the experimental chamber, and the proteins were visualized. For all three conditions, distributions of the stoichiometry of these foci, both overall and within 0.2 µm of the HtH origin, are included as panels (ii) and (iii).

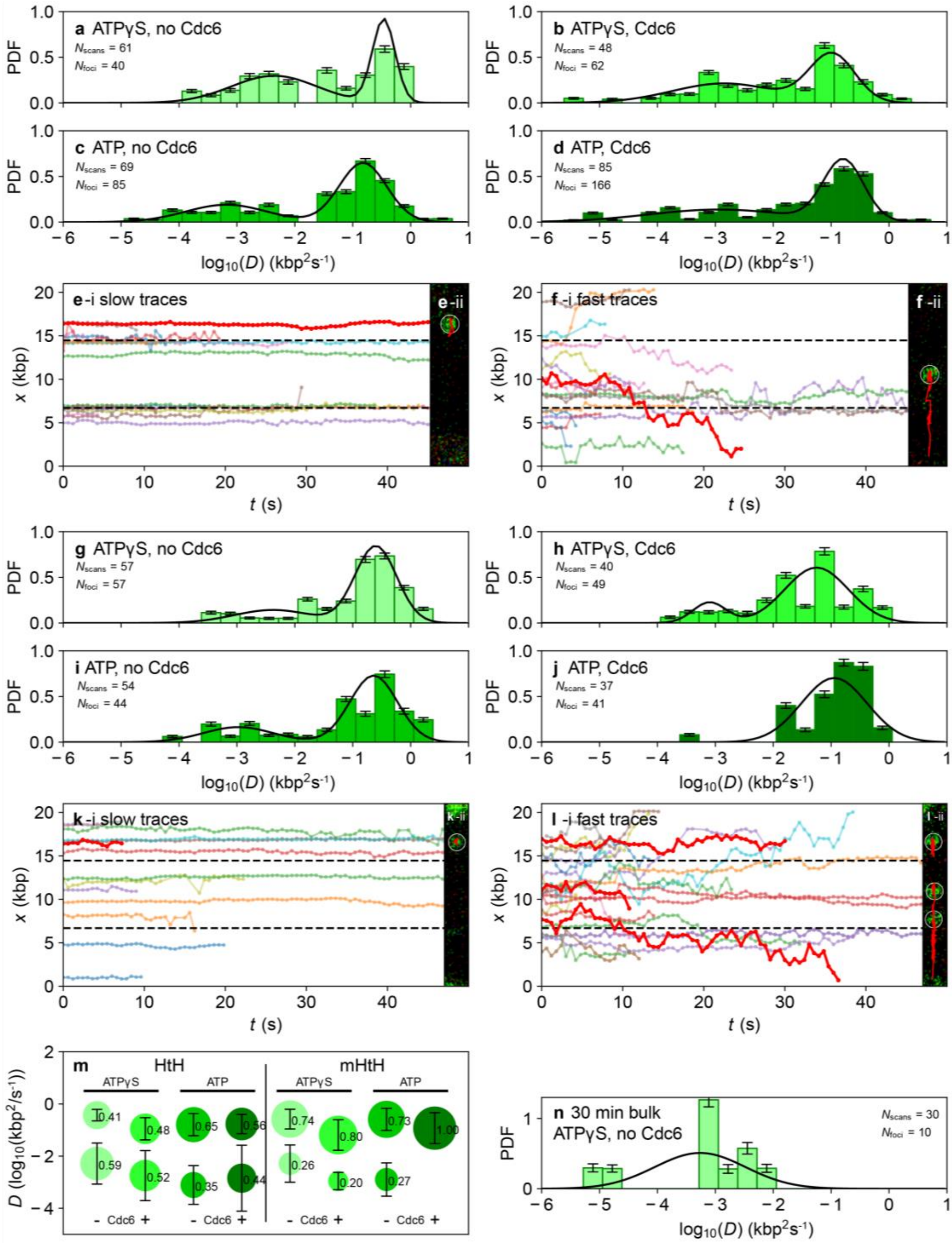

Supplementary Figure 7. The diffusive motion of ORC under different biochemical conditions on DNA containing the HtH or the mHtH origin, and on DNA following a 30 min incubation in bulk.

(a-d) Histograms of the diffusion constants of foci containing 1 or 2 ORC molecules on HtH DNA for the specific conditions: (a) incubation of ORC in ATPγS; diffusion constants (mean ± SEM, in $kbp^2\ s^{-1}$) are 0.06 ± 0.04 and 0.97 ± 0.09 ($p < 0.0001$ by one-way ANOVA); (b) incubation of ORC and Cdc6 in ATPγS; diffusion constants (mean ± SEM, in $kbp^2\ s^{-1}$) are 0.05 ± 0.06 and 0.41 ± 0.06 ($p = 0.0001$ by one-way ANOVA); (c) incubation of ORC in ATP; diffusion coefficients (mean ± SEM, in $kbp^2\ s^{-1}$) are 0.008 ± 0.004 and 0.60 ± 0.07 ($p < 0.0001$ by one-way ANOVA); (d) incubation of ORC and Cdc6 in ATP; diffusion coefficients (mean ± SEM, in $kbp^2\ s^{-1}$) are 0.2 ± 1.4 and 0.56 ± 0.05 ($p = 0.8$ by one-way ANOVA is n.s. due to the width of the slower population). In all, two populations of diffusion constants are found. These populations are fit to lognormal distributions, taking into account the error bars derived from bootstrapping the dataset 100 times. (e,f) Sample time traces drawn from the data sets in (a-d)

showing the motion of JF549-ORC in (e) the slowly diffusive population and (f) the rapidly diffusive population. Molecules are imaged at intervals of 0.6 s until they either dissociate from the DNA (Supplementary Fig. 3) or bleach (Supplementary Fig. 5). (g-j) Histograms of the diffusion constants of ORC on mutated HtH DNA, ordered and analyzed as in panels (a-d). Fitted diffusion coefficients (mean ± SEM, in $kbp^2\ s^{-1}$) are (g) 0.05 ± 0.03 and 0.88 ± 0.09 ($p < 0.0001$ by one-way ANOVA); (h) 0.0034 ± 0.0006 and 0.37 ± 0.09 ($p = 0.04$ by one-way ANOVA); (i) 0.008 ± 0.005 and 1.0 ± 0.1 ($p < 0.0002$ by one-way ANOVA); (j) 0.7 ± 0.2. (k,l) Sample time traces drawn from the data sets in (g-j) showing the motion of JF549-ORC molecules in (k) the slowly diffusive population and (l) the rapidly diffusive population. (m) Overview of the fitted diffusion constants for the conditions tested. (n) Histogram of the diffusion constants of ORC on HtH after a 30-min bulk incubation of ORC in ATPγS. The fitted diffusion coefficient (mean ± SEM) is 0.01 ± 0.01 $kbp^2\ s^{-1}$.

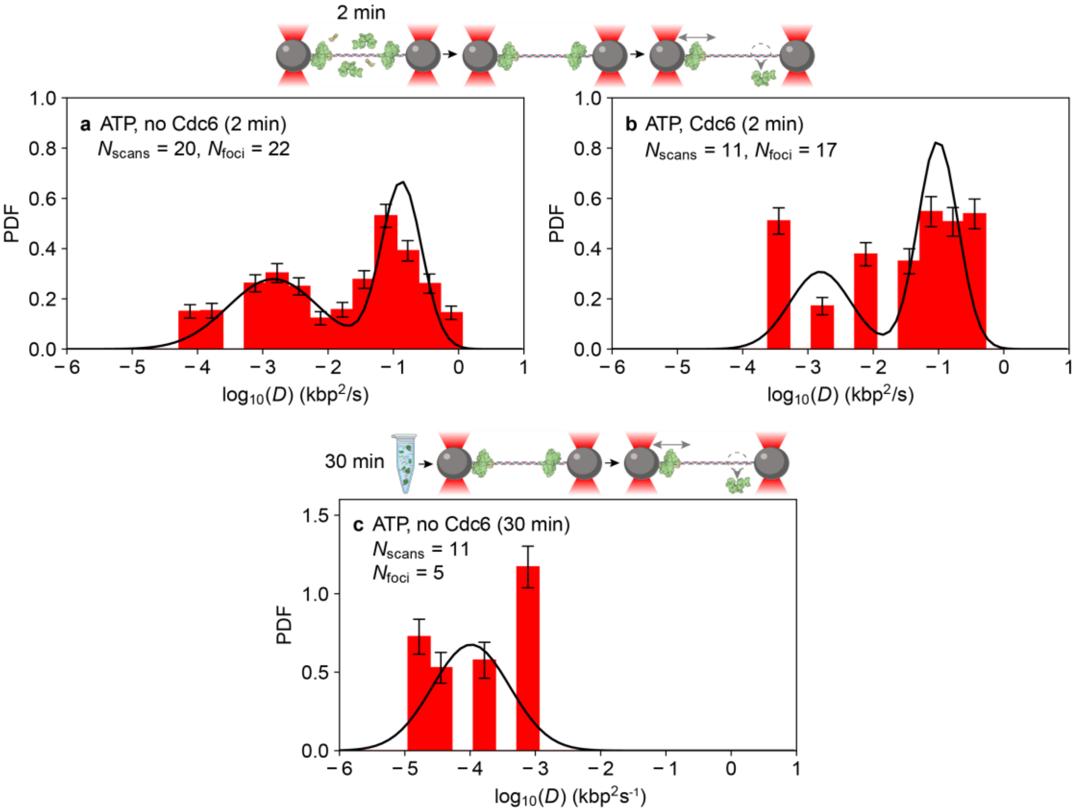

Supplementary Figure 8. The diffusive motion of ORC labeled in red.

(a,b) Histograms of the diffusion constants foci containing 1 or 2 JF646-ORC molecules until they either dissociate from the DNA (Supplementary Fig. 3) or bleach (Supplementary Fig. 5) following a 2-min incubation in the flow cell. Specific conditions are: (a) incubation of ORC in ATP; fitted diffusion coefficients (mean ± SEM) are 0.02 ± 0.02 and 0.48 ± 0.09 kbp^2 s^{-1} ($p<0.0001$ by one-way ANOVA); (b) incubation of ORC and Cdc6 in ATP; fitted diffusion coefficients (mean ± SEM) are 0.010 ± 0.005 and 0.37 ± 0.07 kbp^2 s^{-1} ($p = 0.001$ by one-way ANOVA). (c) Histograms of the diffusion constants of foci containing 1 or 2 JF646-ORC molecules until they either dissociate from the DNA (Supplementary Fig. 4) or bleach (Supplementary Fig. 3) following a 30-min bulk incubation. The fitted diffusion coefficient (mean ± SEM) is 0.0008 ± 0.0006 kbp^2 s^{-1}. In all cases the error bars were derived by bootstrapping the diffusion coefficient distribution 100 times.

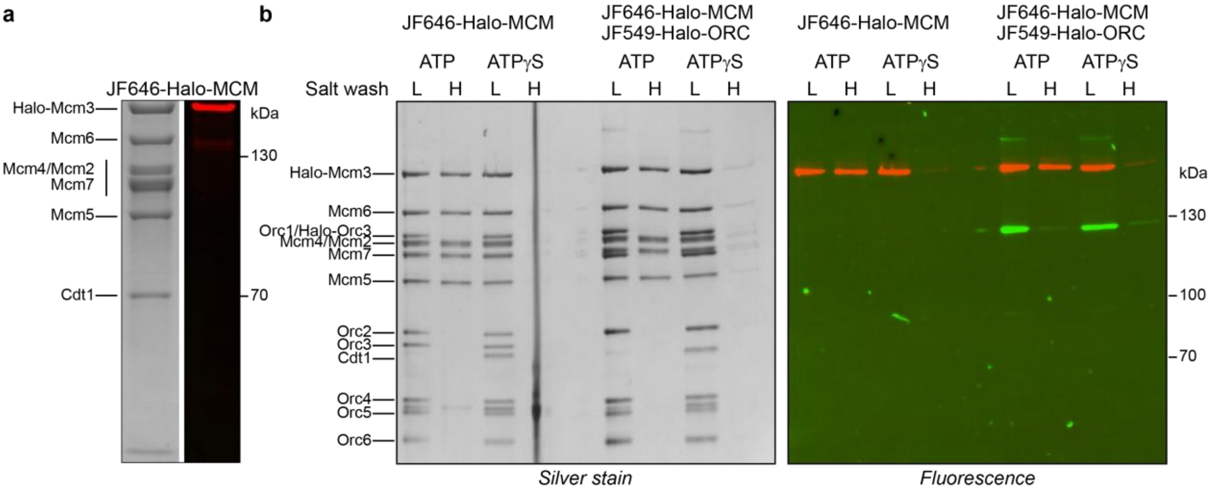

Supplementary Figure 9. Purification of labeled MCM, and loading assay with labeled ORC and labeled MCM.

(a) SDS-PAGE gel of labeled JF646-MCM (estimated labelling efficiency 81%), imaged using (left) silver staining and (right) fluorescence. Uncropped gel images are available as Source_data. (b) Loading assay performed as described in Supplementary Fig. 1, but in the presence of JF646-MCM (first four lanes) and JF549-ORC and JF646-MCM (second four lanes). Products are run on SDS-PAGE gel and imaged using (left) silver staining and (right) fluorescence. Experiments were reproduced at least three times.

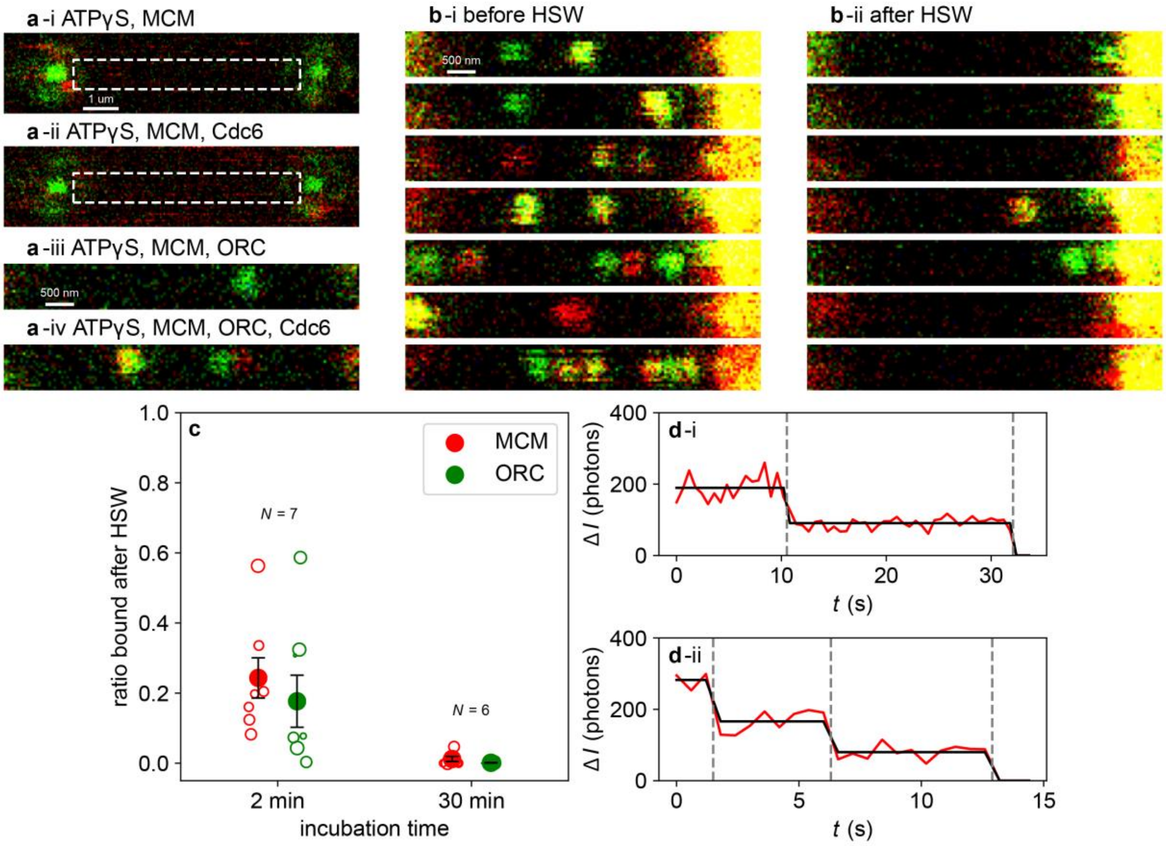

Supplementary Figure 10. Control experiments probing MCM recruitment in ATPγS.

(a) Representative images acquired in buffer containing ATPγS showing the dependence of MCM recruitment on the presence of both ORC and Cdc6. (i) in the absence ORC and Cdc6, no recruitment of MCM is observed (25 DNA visualized, $N_{red\ foci}=0$, $N_{green\ foci}=0$); (ii) in the absence of ORC but the presence of Cdc6, no recruitment of MCM is observed (25 DNA visualized, $N_{red\ foci}=0$, $N_{green\ foci}=0$); (iii) in the presence of ORC and MCM, only ORC is observed to bind to the DNA (25 DNA visualized, $N_{red\ foci}=1$, $N_{green\ foci}=9$); (iv) in the presence of both ORC and Cdc6, binding of ORC and recruitment of MCM are both observed, as expected (25 DNA visualized, $N_{red\ foci}=38$, $N_{green\ foci}=4$). (b) (i) Images showing the presence of both JF549-ORC and JF646-MCM molecules following a 2-min incubation with DNA in the flow cell; (ii) the same DNA molecules imaged following a high-salt wash performed in flow cell. (c) Quantification of the images in panel (b), showing the ratio of the fluorescence emitted by all JF549-ORC (green) and all JF646-MCM molecules (red) before and after the high-salt wash. The same ratio is plotted for an experiment with incubation in bulk for 30 min followed by high-salt wash and visualization in the flow cell. Circles indicate individual data points; filled circles and error bars indicate the mean ± S.D. (d) (i, ii) Counting of the number of bleaching steps of JF646-MCM within a focus permits quantification of the stoichiometry of MCM on the DNA.

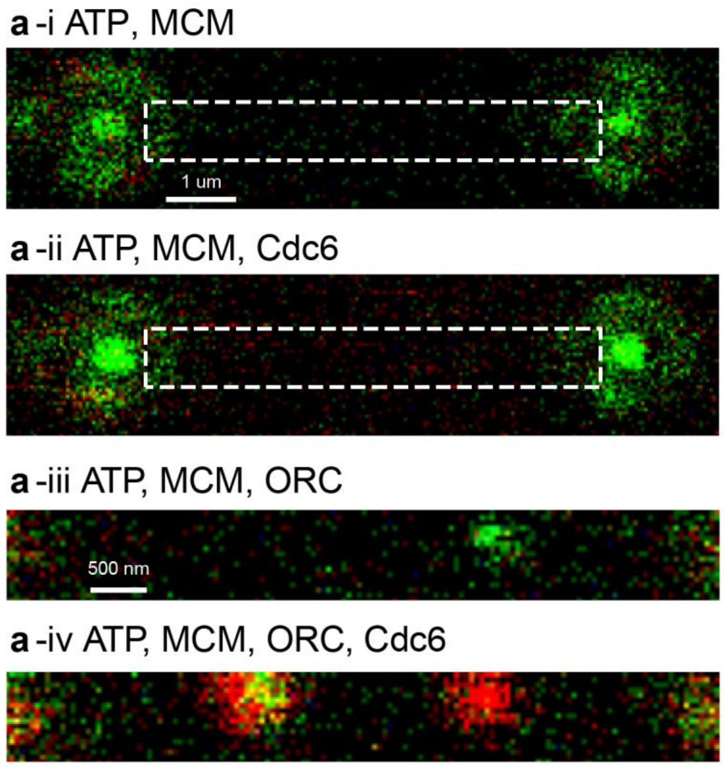

Supplementary Figure 11. Control experiments probing MCM loading in ATP.

(a) Representative images acquired in buffer containing ATP showing the dependence of MCM recruitment on the presence of both ORC and Cdc6. (i) in the absence ORC and Cdc6, no recruitment of MCM is observed (45 DNA visualized, $N_{red\ foci}$=0, $N_{green\ foci}$=0); (ii) in the absence of ORC but the presence of Cdc6, no recruitment of MCM is observed (39 DNA visualized, $N_{red\ foci}$=0, $N_{green\ foci}$=0); (iii) in the presence of ORC (green) and MCM (red), only ORC is observed to bind to the DNA (25 DNA visualized, $N_{red\ foci}$=1, $N_{green\ foci}$=11); (iv) in the presence of both ORC and Cdc6, binding of ORC and recruitment of MCM are both observed, as expected (17 DNA visualized, $N_{red\ foci}$=34, $N_{green\ foci}$=9).

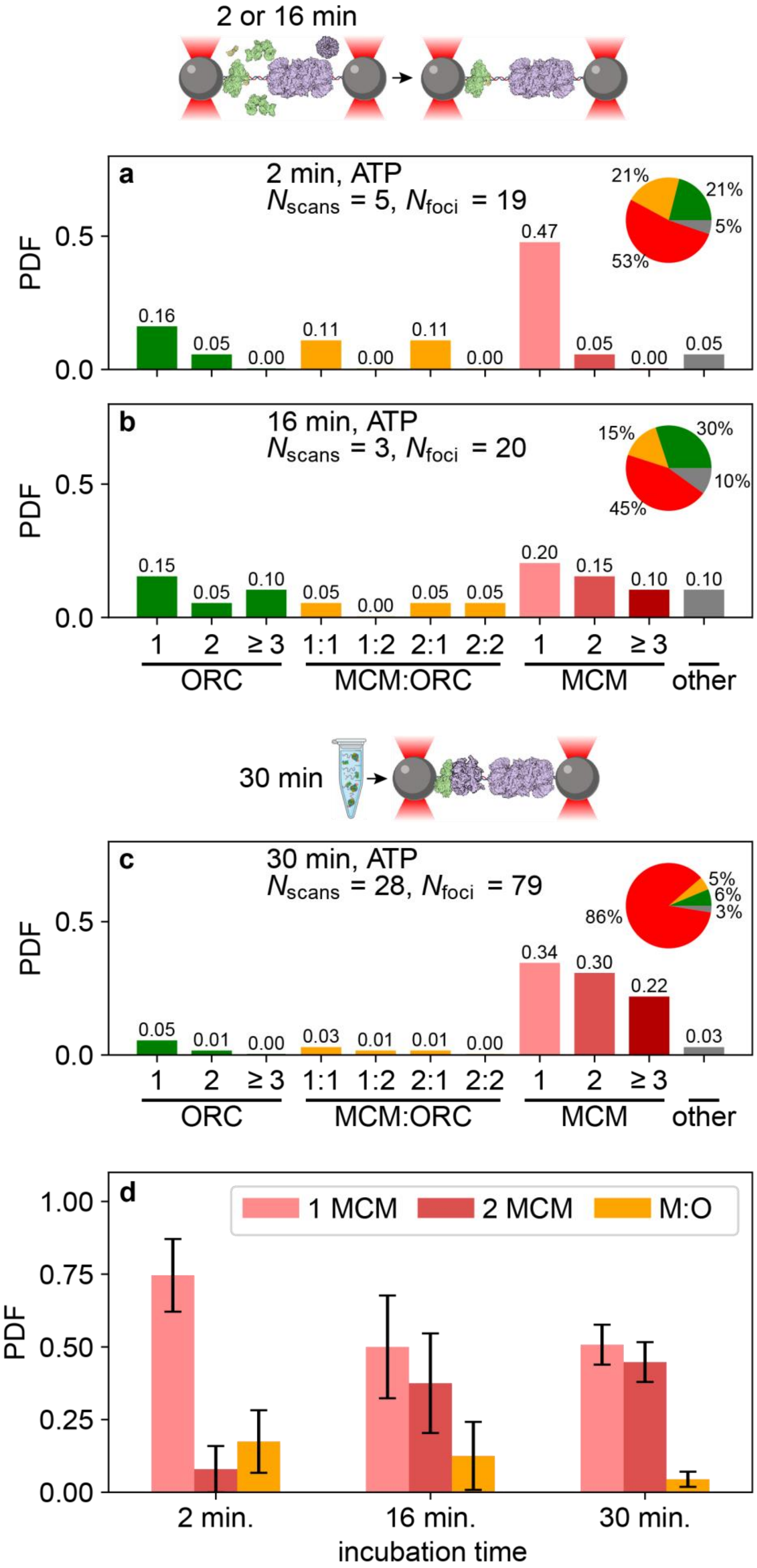
2 or 16 min
a
2 min, ATP
N_{scans} = 5, N_{foci} = 19
21%
21%
5%
53%
PDF
0.5
0.0
0.16
0.05
0.00
0.11
0.00
0.11
0.00
0.47
0.05
0.00
0.05
b
16 min, ATP
N_{scans} = 3, N_{foci} = 20
15%
30%
10%
45%
PDF
0.5
0.0
0.15
0.05
0.10
0.05
0.00
0.05
0.05
0.20
0.15
0.10
0.10
1 2 ≥ 3 1:1 1:2 2:1 2:2 1 2 ≥ 3
ORC
MCM:ORC
MCM
other
30 min
c
30 min, ATP
N_{scans} = 28, N_{foci} = 79
5%
6%
3%
86%
PDF
0.5
0.0
0.05
0.01
0.00
0.03
0.01
0.01
0.00
0.34
0.30
0.22
0.03
1 2 ≥ 3 1:1 1:2 2:1 2:2 1 2 ≥ 3
ORC
MCM:ORC
MCM
other
d
1 MCM
2 MCM
M:O
PDF
1.00
0.75
0.50
0.25
0.00
2 min.
16 min.
30 min.
incubation time

Supplementary Figure 12. Stoichiometry of MCM loaded in ATP prior to HSW.
(a,b) Stoichiometry following a loading reaction including JF549-ORC and JF646-MCM performed in the flow cell in the presence of ATP. The duration of the incubation is (a) 2 min or (b) 16 min. (c) Stoichiometry following a 30-min bulk loading reaction including JF549-ORC and JF646-MCM in the presence of ATP. (d) Relative abundances of single MCM molecules, double MCM molecules, and 1:1 MCM:ORC complexes for the data shown in panels (a)-(c). Error bars represent the error of sample proportion, $p(1-p)/n$, where p is the proportion of a sample in a given population, and n is the sample size.

Supplementary Table 1. Names and sequences of all primers and gBlocks^{TM} gene fragments used in this study.

Primer number	sequence
TL-001	ATACCTCTATACTTTAACGTCAAGGAG
TL-002	AGCGAGATGATAATCCTGTGAGGG
TL-019	CAAGGCTGTGGACATCGGCCCAGGTCTGAATCTGCTGC
TL-020	GCAGCAGATTCAGACCTGGGCCGATGTCCACAGCCTTG
TL-021	TGTTAGTTAGTTACTTAAGCTCG
TL-022	TCGAGTGCGGCCGCGAATTCG
TL-023	GCCCGCGAGACCTTCCAGGCATTCCGCACCACCGACG
TL-024	CGTCGGTGGTGCGGAATGCCTGGAAGGTCTCGCGGGC
TL-025	TCCCCCCGGGGGGAATGGCAGAAATCGGTACTGG
TL-026	TCCCCCCGGGGGGAAGCCGGAAATCTCGAGCGTCG
TL-027	TAAAATGTTGCTGAGGTGAGG
TL-028	TAGCAGCAGAAACAGCAATGAAG
TL-037	TTGATGAATGACACTCCATTGCG
TL-063	ATTAGTTTTTTAGCCTTATTTCTGG
TL-064	TATGATTATTAAACTTCTTTGCG
TL-084	ATGGCAGAAATCGGTACTGG
TL-085	GCCGGAAATCTCGAGCGTCG
TL-086	CCAGTACCGATTTCTGCCATACCTTGGAAGTACAAGTTTTCCAAAG
TL-087	CGACGCTCGAGATTTCCGGCGGTGAAATGGCTAAGACTTTGAAG
TL-119	TATTAAACTTCTTTGCGTCCATCC
TL-120	ACTTCGAACCATCTCAAGTAAGTC
TL-121	AAAATAAACTATTATTTTAGC
TL-122	AAAACGAAGAACAAATTTACG
TL-123	TTTCGTTCAAAGCCTTCATAACG
TL-124	CCAGAAAGACAATTGGGTAACA
TL-125	AGAAGAAAGAGTCGTTCAAACC
TL-126	GTTGAAGACTACTCAAAAGCACC
TL-127	CGAATTCGTTTTCTCTAGCTGG
TL-128	AAGGACAAGGCTACTCAAACTGC
TL-129	AGCCTTGTCCTTCTTTTGTGG
TL-130	TATGAAGAGGAAAAATTGGC
TL-131	TCTCTTTCTCTCTTTTCCTTAATACC
TL-132	AGAATTAAGAAGGACGAAAAGG
TL-133	TTCCAAGAAGTTTCTCTTAGAACC
TL-134	TTACTAGAGAAGAATTCTCTTTGG

**TL-135**	**AACGTACTTAGCACCTTCAGCACC**
**TL-136**	**TTCTTCTGGTGCTGAAGGTGC**
**TL-155**	**GGCGCGCC gaattcCTCGATTTTTTTATGTTTAGTTTCGCG**
**TL-156**	**GGCGCGCC cccgggTCGTGCGC**
**TL-157**	**TCTAAATCTGCACAGCCGAATTGCG**
**TL-169**	**GGCGCGCCgaattcCTCGATTTTTggATGgggAGTTTCG**
**TL-441**	**AAGAAGCTAAGAGAGCTATGAACGAAGACGAAAC**
**TL-442**	**CTCAAAGTTTCGTCTTCGTTCATAGCTCTCTTAG**
**TL-443**	**TAAGGCGCGCCTATAAAACAATGGCTAAGACTTTGAAGGACTTGC**
**TL-446**	**TAAGGCGCGCCTATAAAACAATGAAGAGAAGATGGAAGAAGAACTTCAT**
**TL-447**	**GATTGGTTCAAGTCAGACATGCCGGAAATCTCGAGC**
**TL-449**	**taaccctcactaaagggaacaaaagc**
**TL-450**	**AACTTAGTGTTAGATGGGTCC**
**TL-451**	**AAGTTGAAGTTAGTAATGTTC**
**TL-452**	**TTGGAAAATTTGGTTACCGTAC**
**TL-453**	**AGTAGTTTCAGAGTACAACAAG**
**TL-454**	**TCGCGGTCAACAGGATTCAGG**
**TL-455**	**AATGCAGCGATGGGTCGGTGC**
**TL-456**	**AATTATTAAGCAATCTATTATTC**
**TL-457**	**TCCAGTTAGACAAACTTTGTTG**
**TL-458**	**AATTGTCTAACAACTTGACTGG**
**TL-459**	**tcacgctgcgcgtaaccacc**
**TL-460**	**ATAATTCTTCTCAATTCTTGC**
**TL-461**	**TTGAAAGAACCGTCCTTAGCTC**
**TL-462**	**TCTAGACTTAACTCTCTTTTCC**
**TL-463**	**TGAATTGTTCCTTGTAAGATTG**
**TL-464**	**TGTTGGTCCGCGCGATGGCACC**
**TL-465**	**GAACGTTTTTATCGAGGGTAC**
**TL-466**	**AAGATGAACGTTTCTGAATTCG**
**TL-467**	**AAGTTGTACTCTGCTGCTGACGC**
**TL-468**	**TAACTTGTCTAACATTGAAAAG**
**TL-469**	**AACAGAGGTTTGGAAGAATTC**
**TL-470**	**AAGTTGTACAGAGAAGCTAAC**
**TL-472**	**CGACGCTCGAGATTTCCGGCATGTCTGACTTGAACCAATCTAAGAAGATGAACG**
**TL-473**	**TTCTTCCATCTTCTCTTCATTGTTTTATAGGCGCGCCTTATATTGAATTTTC**

gBlocks™ gene fragments	Sequence
**pGC203**	**gaattcCTCGATTTTTTATGTTTAGTTTCGCGGACGACGGTTTCGAGG<br>TGGCGGTCTGGACCACGCCGGAGAGCGTCGAAGCGGAGGCGGTGT<br>TCGCCGAGATCGGCTCGCGCATGGCCGAGTTGAGCGGTTCCAGGCT<br>GGCCAAACAGCATCAGATGGAACTAAACATAAAAAtACACCGGCCT<br>CA<br>GCATCCGGTACCTCAGCTGGCCACCGTCGGCGTCTCGCACGACCACC<br>AGTGCAAGGGTCTGAGCAGCGCCGTCGTGCTCCTCGGAGTGGAGG<br>CA<br>GCCGAGCGCGACGGTGTGCCCGCCTTCCTGGAGACCTCCGCGCTCC<br>GCAACCTCCACTTCTACGAGCGGCTCGGCTTCACCGTCACCGCCGAC<br>G<br>TCGAGGTGCCCGAAGGACCGCGCACCTGGTGCATGACCCGCAAGCC<br>CGGTGCCTGACGCTCGCCACACGACCCGCAGCGCCCGACCGAAAGG<br>AG<br>CGCACGAcccggg**
**pGC218**	**gaattcCTCGATTTTTggATGgggAGTTTCGCGGACGACGGTTTCGAGG<br>TGGCGGTCTGGACCACGCCGGAGAGCGTCGAAGCGGAGGCGGTGT<br>TCGCCGAGATCGGCTCGCGCATGGCCGAGTTGAGCGGTTCCAGGCT<br>GGCCAAACAGCATCAGATGGAACTcccCATAccAAtACACCGGCCTC<br>A<br>GCATCCGGTACCTCAGCTGGCCACCGTCGGCGTCTCGCACGACCACC<br>AGTGCAAGGGTCTGAGCAGCGCCGTCGTGCTCCTCGGAGTGGAGG<br>CA<br>GCCGAGCGCGACGGTGTGCCCGCCTTCCTGGAGACCTCCGCGCTCC<br>GCAACCTCCACTTCTACGAGCGGCTCGGCTTCACCGTCACCGCCGAC<br>G<br>TCGAGGTGCCCGAAGGACCGCGCACCTGGTGCATGACCCGCAAGCC<br>CGGTGCCTGACGCTCGCCACACGACCCGCAGCGCCCGACCGAAAGG<br>AG<br>CGCACGAcccggg**

Supplementary References

1. Liachko, I., Youngblood, R. A., Keich, U. & Dunham, M. J. High-resolution mapping, characterization, and optimization of autonomously replicating sequences in yeast. *Genome Res.* **23**, 698–704 (2013).
2. Coster, G. & Diffley, J. F. X. Bidirectional eukaryotic DNA replication is established by quasi-symmetrical helicase loading. *Science* **357**, 314–318 (2017).
