# Supplementary material for: DNA replication origins retain mobile licensing proteins
Source: Nat Commun. 2021 Mar 26;12:1908. doi: 10.1038/s41467-021-22216-x (PMC7998030; doi:10.1038/s41467-021-22216-x)
Supplement: Supplementary file 2 — Description of Additional Supplementary Files [file 41467_2021_22216_MOESM2_ESM.pdf]

Description of Additional Supplementary Files

File Name: Supplementary Movie 1
Description: Slowly diffusive motion of JF549-ORC on 21.2 kbp DNA (1 frame every 0.6 s).

File Name: Supplementary Movie 2
Description: Rapidly diffusive motion of JF549-ORC on 21.2 kbp DNA (1 frame every 0.6 s).

File Name: Supplementary Movie 3
Description: Characteristic diffusive motion of colocalized JF549-ORC and JF646-MCM on 21.2 kbp DNA in buffer containing ATPS (1 frame every 0.6 s).

File Name: Supplementary Movie 4
Description: Characteristic diffusive motion of foci containing either 1 or 2 JF646-MCM on 21.2 kbp DNA following loading in buffer containing ATP followed by a HSW (1 frame every 0.6 s).

File Name: Supplementary Movie 5
Description: Characteristic diffusive motion of foci containing either 1 or 2 JF646-MCM on 21.2 kbp DNA following loading in buffer containing ATP followed by a HSW (1 frame every 120 s).
