# Supplementary material for: DNA replication origins retain mobile licensing proteins
Source: Nat Commun. 2021 Mar 26;12:1908. doi: 10.1038/s41467-021-22216-x (PMC7998030; doi:10.1038/s41467-021-22216-x)
Supplement: Supplementary file 8 — Reporting Summary [file 41467_2021_22216_MOESM8_ESM.pdf]

nature research

Corresponding author(s): John F.X. Diffley, Nynke Dekker

Last updated by author(s): Feb 23, 2021

Reporting Summary

Nature Research wishes to improve the reproducibility of the work that we publish. This form provides structure for consistency and transparency in reporting. For further information on Nature Research policies, see our Editorial Policies and the Editorial Policy Checklist.

Statistics

For all statistical analyses, confirm that the following items are present in the figure legend, table legend, main text, or Methods section.

n/a	Confirmed	
☐	☒	The exact sample size (*n*) for each experimental group/condition, given as a discrete number and unit of measurement
☐	☒	A statement on whether measurements were taken from distinct samples or whether the same sample was measured repeatedly
☐	☒	The statistical test(s) used AND whether they are one- or two-sided<br>*Only common tests should be described solely by name; describe more complex techniques in the Methods section.*
☒	☐	A description of all covariates tested
☒	☐	A description of any assumptions or corrections, such as tests of normality and adjustment for multiple comparisons
☐	☒	A full description of the statistical parameters including central tendency (e.g. means) or other basic estimates (e.g. regression coefficient) AND variation (e.g. standard deviation) or associated estimates of uncertainty (e.g. confidence intervals)
☐	☒	For null hypothesis testing, the test statistic (e.g. *F*, *t*, *r*) with confidence intervals, effect sizes, degrees of freedom and *P* value noted<br>*Give P values as exact values whenever suitable.*
☒	☐	For Bayesian analysis, information on the choice of priors and Markov chain Monte Carlo settings
☒	☐	For hierarchical and complex designs, identification of the appropriate level for tests and full reporting of outcomes
☒	☐	Estimates of effect sizes (e.g. Cohen's *d*, Pearson's *r*), indicating how they were calculated

Our web collection on statistics for biologists contains articles on many of the points above.

Software and code

Policy information about availability of computer code

Data collection	Data was collected using the commercial software provided with the instrument: Lumicks Bluelake version 1.6.13
Data analysis	We used the open-source single-particle tracking ImageJ plugin TrackMate v5.2.0 for spot detection, Python 3.8 for subsequent analysis (with analysis libraries numpy 1.19.0, scipy 1.5.0, scikit-image 0.17.2, scikit-learn 0.23.1, pandas 1.05). The analysis of the acquired data was performed using custom-written scripts which are available upon request.

For manuscripts utilizing custom algorithms or software that are central to the research but not yet described in published literature, software must be made available to editors and reviewers. We strongly encourage code deposition in a community repository (e.g. GitHub). See the Nature Research guidelines for submitting code & software for further information.

Data

Policy information about availability of data

All manuscripts must include a data availability statement. This statement should provide the following information, where applicable:

- Accession codes, unique identifiers, or web links for publicly available datasets
- A list of figures that have associated raw data
- A description of any restrictions on data availability

The data that support the findings of this study are available from the corresponding author upon request.

Field-specific reporting

Please select the one below that is the best fit for your research. If you are not sure, read the appropriate sections before making your selection.

☒ Life sciences ☐ Behavioural & social sciences ☐ Ecological, evolutionary & environmental sciences

For a reference copy of the document with all sections, see nature.com/documents/nr-reporting-summary-flat.pdf

Life sciences study design

All studies must disclose on these points even when the disclosure is negative.

Sample size	Sample size is mentioned in the figure for each experiment, and the results of the quantitative analysis of multiple DNA tethers are presented as histograms. No sample size precalculation was performed, but data collection continued until the data set was large enough to perform meaningful statistical analysis of the distribution. This typically required at least 20 individual DNA tethers containing at least 50 fluorescent foci. Sample size is conditioned by the intrinsic low throughput of the optical force/fluorescence instrument, and was selected as a reasonable minimum to perform statistical analysis. A small number of control experiments whose throughput was more limited (due to e.g. long pre-incubation times) was associated with a number of fluorescent foci below 50.
Data exclusions	We exclude localizations more than 10 kb from the center of the DNA, which correspond to false positives from fluorescent proteins adhered to the optically trapped beads. These edge effects account for the majority of bright "noise" foci, but we also exclude foci elsewhere on the DNA with an intensity > 10x the single-fluorophore intensity, which represent protein aggregation. The noise foci occur in all of our single-molecule experiments, and every data set shown in this paper has had noise foci excluded. Depending on the data set, the number of noise foci ranged from 0% to 11% of all the foci originally detected.
Replication	When collecting a data set with single-molecule optical force spectroscopy, every DNA-protein complex is trapped and measured independently. For each experimental condition, these independent measurements are repeated as many times as needed to reach the statistics discussed above in "Sample Size," with all other experimental conditions held constant. Even data sets that were collected over the course of one day are the result of dozens to hundreds of individual experiments performed in identical fashion on identically-prepared DNA-protein systems.
Randomization	Apart from data exclusion process described before, no other experimental groups were further introduced.
Blinding	These experiments were not blinded, but the negative controls are described in the text and supplement. In general, blinding is not feasible in single-molecule experiments, and targeted controls are preferred.

Reporting for specific materials, systems and methods

We require information from authors about some types of materials, experimental systems and methods used in many studies. Here, indicate whether each material, system or method listed is relevant to your study. If you are not sure if a list item applies to your research, read the appropriate section before selecting a response.

Materials & experimental systems

n/a	Involved in the study	
☒	☐	Antibodies
☒	☐	Eukaryotic cell lines
☒	☐	Palaeontology and archaeology
☒	☐	Animals and other organisms
☒	☐	Human research participants
☒	☐	Clinical data
☒	☐	Dual use research of concern

Methods

n/a	Involved in the study	
☒	☐	ChIP-seq
☒	☐	Flow cytometry
☒	☐	MRI-based neuroimaging
