# Supplementary material for: DNA replication origins retain mobile licensing proteins
Source: Nat Commun. 2021 Mar 26;12:1908. doi: 10.1038/s41467-021-22216-x (PMC7998030; doi:10.1038/s41467-021-22216-x)
Supplement: Supplementary file 9 — Source Data [file 41467_2021_22216_MOESM9_ESM.pdf]

JF549-Halo-ORC

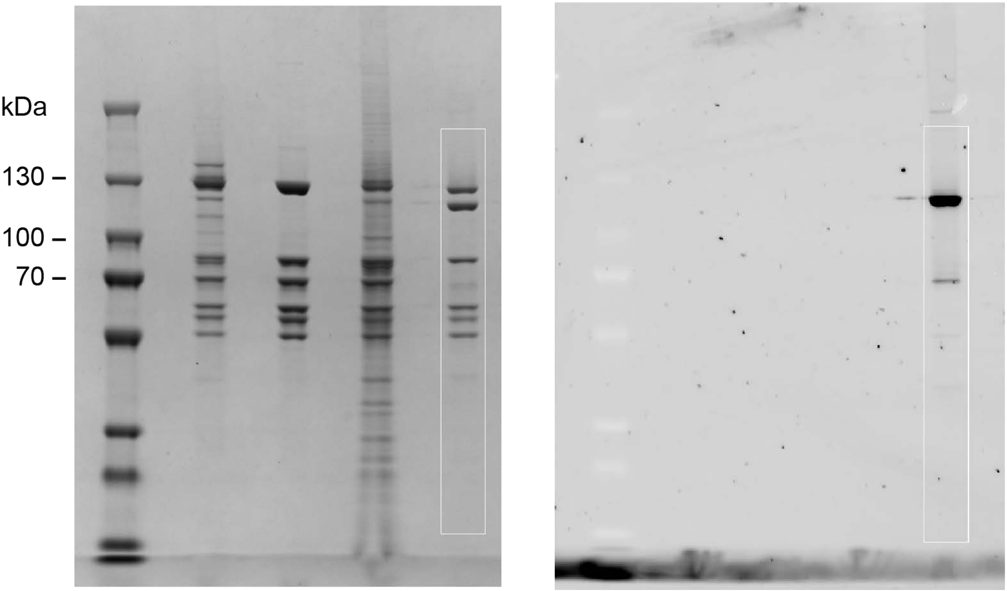


Full scans Supplementary Figure 1.2c: rectangules correespond to samples included in the manuscript. Remaining samples not included in this study.

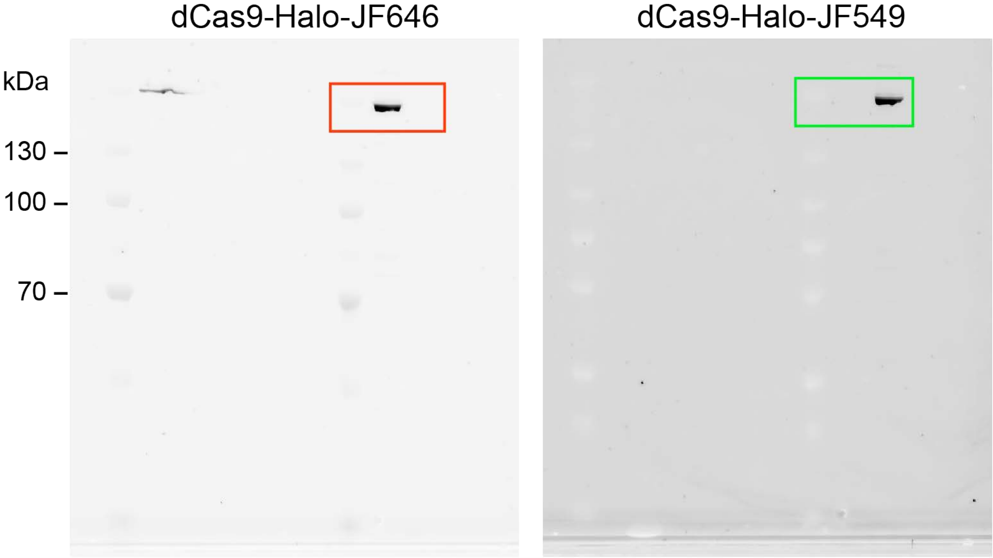


Full scans Supplementary Figure 1.5a: rectangules correespond to samples included in the manuscript. Remaining samples not included in this study.

JF646-Halo-MCM

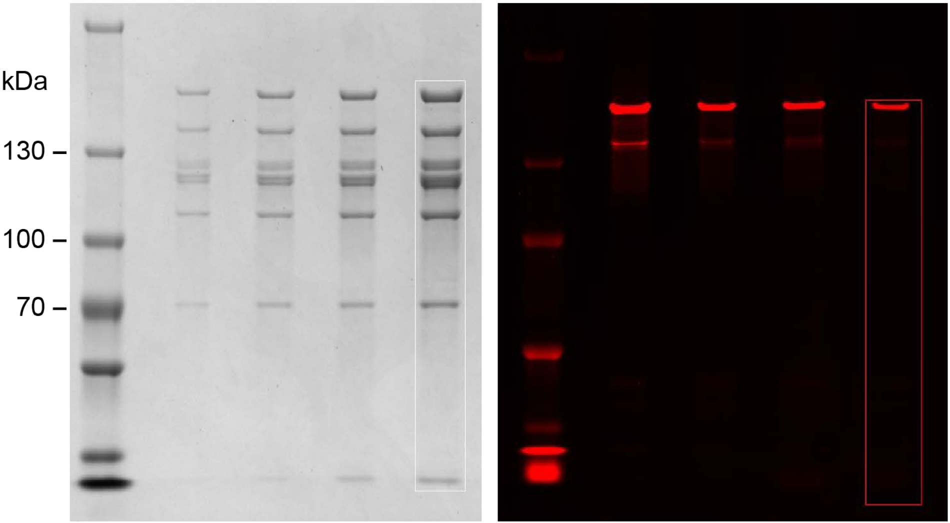


Full scans Supplementary Figure 3.1a: rectangules correespond to samples included in the manuscript. Remaining samples not included in this study.
